# Mitochondrial DNA Reveals Genetic Structuring of *Pinna nobilis* across the Mediterranean Sea

**DOI:** 10.1371/journal.pone.0067372

**Published:** 2013-06-28

**Authors:** Daria Sanna, Piero Cossu, Gian Luca Dedola, Fabio Scarpa, Ferruccio Maltagliati, Alberto Castelli, Piero Franzoi, Tiziana Lai, Benedetto Cristo, Marco Curini-Galletti, Paolo Francalacci, Marco Casu

**Affiliations:** 1 Dipartimento di Scienze della Natura e del Territorio - Sezione di Zoologia, Archeozoologia e Genetica, Università di Sassari, Sassari, Italy; 2 Dipartimento di Medicina Veterinaria - Sezione di Anatomia, Università di Sassari, Sassari, Italy; 3 Dipartimento di Biologia, Università di Pisa, Pisa, Italy; 4 Dipartimento di Scienze Ambientali, Informatica e Statistica, Università Cà Foscari, Venezia, Italy; Australian Museum, Australia

## Abstract

*Pinna nobilis* is the largest endemic Mediterranean marine bivalve. During past centuries, various human activities have promoted the regression of its populations. As a consequence of stringent standards of protection, demographic expansions are currently reported in many sites. The aim of this study was to provide the first large broad-scale insight into the genetic variability of *P. nobilis* in the area that encompasses the western Mediterranean, Ionian Sea, and Adriatic Sea marine ecoregions. To accomplish this objective twenty-five populations from this area were surveyed using two mitochondrial DNA markers (COI and 16S). Our dataset was then merged with those obtained in other studies for the Aegean and Tunisian populations (eastern Mediterranean), and statistical analyses (Bayesian model-based clustering, median-joining network, AMOVA, mismatch distribution, Tajima’s and Fu’s neutrality tests and Bayesian skyline plots) were performed. The results revealed genetic divergence among three distinguishable areas: (1) western Mediterranean and Ionian Sea; (2) Adriatic Sea; and (3) Aegean Sea and Tunisian coastal areas. From a conservational point of view, populations from the three genetically divergent groups found may be considered as different management units.

## Introduction

The assessment of genetic variation in marine species and the evaluation of connectivity among populations are crucial components for conservation purposes and resource management [1], [2]. However, direct estimates of dispersal are difficult to obtain for marine organisms [3] because of the multiple factors (e.g., pelagic larval duration, larval phylopatry, regional and local water circulation, and habitat specificity) that influence dispersal capability [4]. Molecular tools are often employed to make indirect inferences about the levels of connectivity among natural populations [5], [6]. Molecular approaches can be particularly useful for determining the influence of such factors as hydrodynamic conditions [7], [8], habitat specificity [9], [10] and various ecological parameters (e.g., [11], [12]) on gene flow and spatial genetic structure. However, the spatial genetic structure of a species may reflect not only its current dispersal capability but also its phylogeographic history. For example, the occurrence of different genetic lineages in Mediterranean species has been explained as the result of either the present dispersal capability or past geomorphologic processes or both (see [13] and references therein). Furthermore, human activities (e.g., intense exploitation, industrialisation, and coastal tourism) often lead to significant reduction of population size, which may result in an increased magnitude of genetic drift.

The fan mussel *Pinna nobilis* (Linnaeus, 1758) (Mollusca: Bivalvia) is an endemic Mediterranean species of great conservation interest that can be regarded as a flag species *sensu* Walpole and Leader-Williams [14] and Heywood [15]. As one of the largest bivalves, with maximum lengths in excess of 1 m, this popular species is able to capture the public imagination [16]. *Pinna nobilis* occurs at depths between 0.5 and 60 m in soft bottoms primarily characterised by seagrass meadows [16]. Its veliger-stage larvae drift in the water column [17] before settling in the sediment and anchoring via a byssus [18]. Although De Gaulejac and Vicente [19] hypothesised a period of 5–10 days, little is known about the length of the larval life cycle of *P. nobilis*. Moreover, Peharda and Vilibić [20] postulated that *P. nobilis* veliger might exhibit daily vertical migration, spending the daylight hours in darker, deeper waters and occupying superficial layers at night, as observed for other bivalves [21].

As a result of the population declines associated with human activity (primarily the harvesting of byssus for the manufacture of so-called “sea silk” and shell collecting [22], [23], secondarily the use as food of the abductor muscle in some Mediterranean regions [24], and the collection of the little, with poor commercial value, pearls [25]), *P. nobilis* has been included in the list of Mediterranean endangered species (Annex IV of the Habitat Directive and Annex II of the Barcelona Convention) since 1995. Remarkably, it is likely that some of the known geographic and ecological barriers [26], [27], [28], which define different marine ecoregions and biogeographic sectors in the Mediterranean [29], [30], may affect the degree of connectivity among *P. nobilis* populations. In the Mediterranean region, several authors have documented population subdivision in relation to physical barriers in other marine species with a high dispersal potential (e.g., [13], [31], [32]).

Despite the conservation importance of *P. nobilis*, few studies have investigated the biology and ecology of this species (e.g., [20], [23], [33], [34]), and fewer still have studied its population genetics. To date, only two molecular surveys have been conducted, one in the Aegean Sea [35] and one along the Tunisian coasts [36]. The former [35] surveyed the genetic variability of four Aegean populations by means of the mitochondrial DNA (mtDNA) markers Cytochrome c Oxidase subunit I (COI) and 16S ribosomal DNA (16S). A high level of haplotypic diversity was found for the COI gene, whereas the 16S gene showed a lower level of variability. These results suggested the lack of genetic structuring among the Aegean populations. In the second study [36], the authors used COI sequences to describe the genetic variability of five populations from the northern, eastern, and southern Tunisian coasts. A North-East decreasing gradient of genetic variability was found among these populations, which was explained in light of the variance in the hydrodynamic regime of the areas analysed.

Knowledge of the amount of genetic variability and distribution in space and time is crucial for a correct diagnosis of the conservation status and viability of populations and the threats to them [37], [38]. Accordingly, it is evident that a molecular analysis performed on a larger number of Mediterranean *P. nobilis* populations could provide a deeper insight into the possible influence of environmental or anthropogenic stress on the population dynamics and evolutionary history of this endangered species, allowing the development of effective conservation measures. Based on this background, the present study was aimed to investigate the large-scale patterns of spatial genetic variation of *P. nobilis* in the Mediterranean. Our primary focus was the appraisal of the genetic structuring and population connectivity of *P. nobilis* in the western Mediterranean, Ionian Sea, and Adriatic Sea ecoregions. We surveyed populations from the Sardinian-Corsican region, Elba Island, Sicily, and the Venetian Lagoon and compared these populations with the available data from the Aegean Sea [35] and Tunisian coasts [36]. This large area is characterised by a complex and variable pattern of marine circulation (e.g., [39]) and encompasses several biogeographic sectors [28]. To accomplish the objective of the present study, we employed two mitochondrial markers (COI and16S) previously used for the investigation of Aegean [35] and Tunisian populations [36].

## Materials and Methods

### Ethics Statement

No field studies involving impacting manipulation, dislocation, or removal of *Pinna nobilis* individuals were performed. For each location under protection all necessary permits were obtained for the sampling activities by the authority responsible for each protected area:

- Gianfranco Russino, Director of the Marine Protected Area of Capo Caccia-Isola Piana (year 2010); location: Baia di Porto Conte;

- Augusto Navone, Director of the Marine Protected Area of Tavolara-Punta Coda Cavallo (year 2010); locations: Molara, Monte Petrosu;

- Mauro Gargiulo, Director of the National Park Arcipelago di La Maddalena (year 2011); location La Maddalena;

- Lorenzo Mascia, Director of the Marine Protected Area of Penisola del Sinis-Isola di Mal di Ventre (year 2011); locations: Oristano, Isola di Mal di Ventre;

- Maddy Cancemi, Director of the Réserve Naturelle des Bouches de Bonifacio (year 2011); location: Isola Piana.

No specific permissions were required for the locations placed out of protected areas, since those locations are not privately-owned or protected in any way.

### Sampling

**Figure 1:**
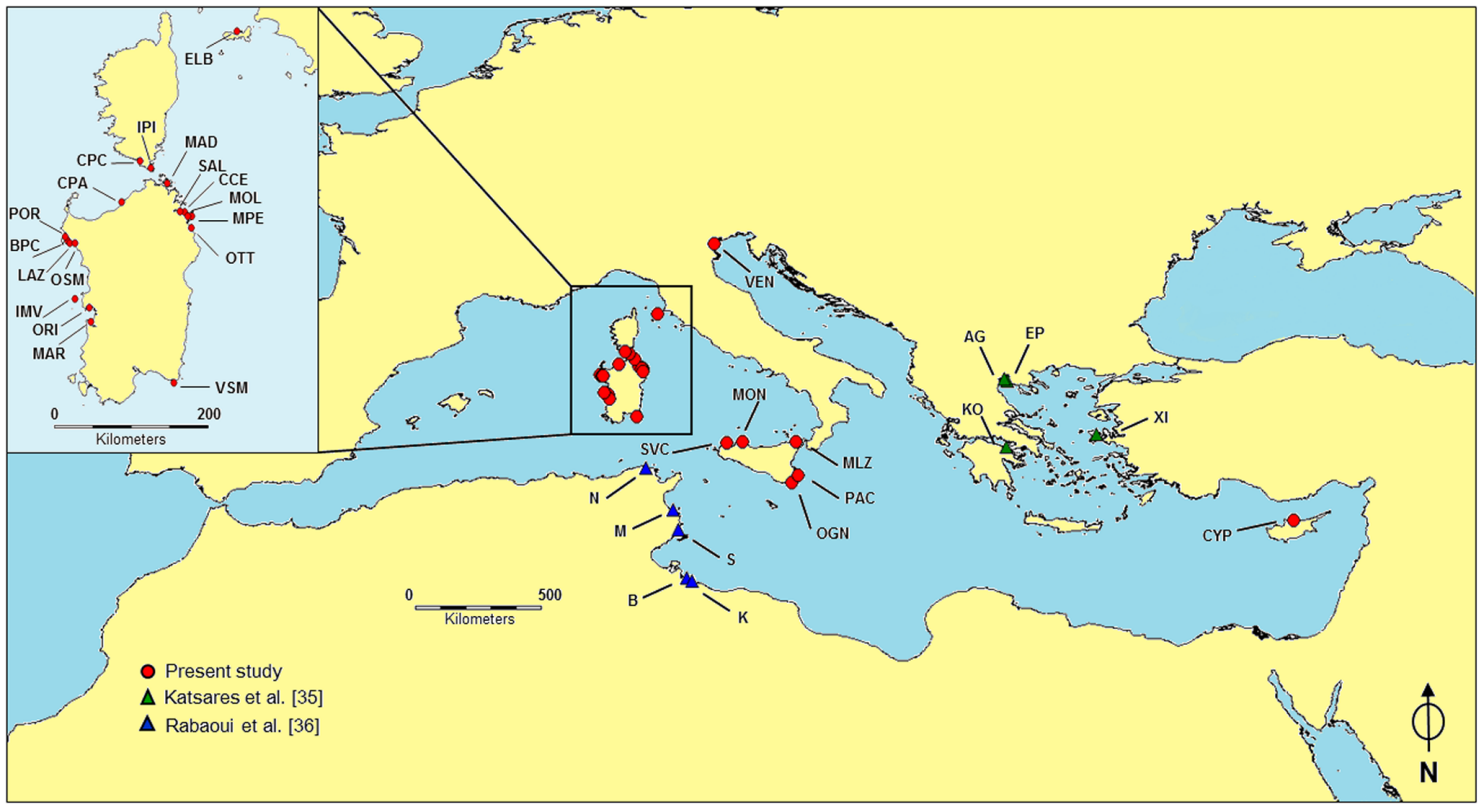
Mediterranean sampling plan. Map of the Mediterranean indicating the localisation of the sampling sites. The relative geographic coordinates are reported in Table 1. The populations are labelled as reported in Table 1.

Overall, we sampled 236 specimens of *P. nobilis* from 25 locations (Table 1 and Figure 1), sited in the following ecoregions according to Spalding et al. [30]: (1) the central part of the western Mediterranean (the Sardinian Sea, the North Tyrrhenian Sea, the South Tyrrhenian Sea, and the Strait of Sicily); (2) the Ionian Sea (the South-East of Sicily); and (3) the Adriatic Sea (the Venetian Lagoon). Two individuals from the Levantine Sea (Cyprus) were also analysed as the easternmost Mediterranean outliers. We developed a specific non-lethal sampling method, performed by SCUBA divers, which does not cause significant damages to the shell and soft tissues of *P. nobilis*. The valves of a given individual were held open with a wooden stick (diameter = 0.5 cm), put in proximity (4–5 cm) of the hinge ligament, and a 20–50 mg sample of mantle tissue was excised using Hartman Alligator Forceps 3.5. The stick was then removed, and the tissue sample stored in a 5 ml tube. The tissue sample was then transferred to the laboratory in a refrigerated box and there preserved in 75% ethanol. This method ensures the survival of the sampled individuals.

**Table 1:** Data collection.

Site	PA	Label	SP	COI	16S	Coordinates
**Sardinia**						
Baia di Porto Conte	yes	BPC	Summer 2010	18	9	40°36′28.56"N; 08°12′40.32′′E
Torre del Porticciolo	no	POR	Summer 2010	3	2	40°38′34.56′′N; 08°11′13.80′′E
Lazzaretto	no	LAZ	Summer 2010	2	2	40°34′40.08′′N; 08°14′51.96′′E
Ospedale Marino	no	OSM	Summer 2010	21	21	40°34′44.90′′N; 08°18′26.81′′E
Molara	yes	MOL	Autumn 2010	11	10	40°51′46.26′′N; 09°43′21.96′′E
Capo Ceraso	no	CCE	Autumn 2010	13	12	40°54′26.10′′N; 09°38′13.62′′E
Le Saline	no	SAL	Spring 2011	5	5	40°54′48.18′′N; 09°35′00.61′′E
Monte Petrosu (Sassi piatti and Isola Cava)	yes	MPE	Autumn 2010	4	4	40°51′56.11′′N; 09°40′23.08′′E
Porto Ottiolu	no	OTT	Spring 2011	5	5	40°44′16.50′′N; 09°42′54.22′′E
Oristano (Sa Mardini and Torre Vecchia)	yes	ORI	Spring 2011	10	10	39°53′38.10′′N; 08°28′58.00′′E
Marceddì	no	MAR	Spring 2011	5	5	39°44′43.00′′N; 08°29′54.30′′E
Isola di Mal di Ventre	yes	IMV	Spring 2011	4	4	39°59′14.10′′N; 08°18′27.50′′E
Villasimius (Capo Caterina)	no	VSM	Summer 2011	4	4	39°06′16.07′′N; 09°30′07.29′′E
Costa Paradiso	no	CPA	Spring 2011	5	5	41°00′43.43′′N; 08°52′22.45′′E
Isola di La Maddalena (Cala Camiciotto)	yes	MAD	Summer 2011	18	18	41°12′41.41′′N; 09°25′42.10′′E
**Corsica**						
Isola Piana	yes	IPI	Autumn 2011	13	15	41°22′33.90′′N; 09°13′47.73′′E
Cala Pesciu Cane	no	CPC	Autumn 2011	12	13	41°26′′47.25′′N; 09°05′41.79′′E
**Elba Island**						
Capo Enfola	no	ELB	Spring 2011	10	10	42°49′25.41′′N; 10°16′07.80′′E
**Sicily**						
San Vito lo Capo (Secca di Cala Rossa)	no	SVC	Spring 2011	7	7	38°09′36.10′′N; 12°46′17.71′′E
Mondello	no	MON	Spring 2011	11	11	38°12′12.00′′N; 13°19′47.12′′E
Milazzo	no	MLZ	Spring 2011	10	9	38°13′01.72′′N; 15°14′43.53′′E
Pachino (Capo Passero)	no	PAC	Spring 2011	8	8	36°42′30.05′′N; 15°07′33.92′′E
Ognina di Siracusa	no	OGN	Spring 2011	15	15	36°58′54.69′′N; 15°16′47.96′′E
**Venetian Lagoon**						
Ottagono Alberoni and Santa Maria del Lago	no	VEN	Spring 2011	20	20	45°21′48.21′′N; 12°19′17.17′′E
**Cyprus**						
Karaoglanoglu	no	CYP	Summer 2011	2	2	35°20′54.52′′N; 33°15′17.16′′E
Aegean Sea*^1^						
Epanomi	–	EP	–	9	8	40°23′13.00′′N; 22°54′02.00′′E
Aggeloyesori	–	AG	–	9	9	40°29′12.00′′N; 22°49′01.00′′E
Xios Island	–	XI	–	5	5	38°29′09.00′′N; 26°08′13.00′′E
Korinthiakos Gulf	–	KO	–	3	3	38°00′10.00′′N; 22°52′45.00′′E
Tunisian coasts*^2^						
Bizerta Lagoon	–	N	–	7	–	37°14′46.91′′N; 09°51′09.25′′E
Monastir (Stah Jaber)	–	M	–	9	–	35°45′20.50′′N; 10°50′03.05′′E
Kerkennah Island	–	S	–	7	–	35°01′40.67′′N; 11°00′44.24′′E
El Bibane Lagoon	–	B	–	9	–	33°16′06.17′′N; 11°18′41.49′′E
El Ketef	–	K	–	17	–	33°10′52.21′′N; 11°29′35.89′′E

Asterisks (*) and superscript numbers identify samples whose sequences were taken from the GenBank database: (1) Katsares et al. [35]; (2) Rabaoui et al. [36].

Sampling period, sample sizes and geographic coordinates for populations of *Pinna nobilis* belonging to the 34 Mediterranean sites here considered. PA: area under protection; SP: sampling period.

### DNA Extraction and PCR

DNA was isolated using the Qiagen DNeasy tissue kit. Mitochondrial regions were amplified using specific primers for COI (L: 5′-GGTTGAACTATHTATCCNCC-3′ and H: 5′-GAAATCATYCCAAAAGC-3′) and 16S (L: 5′-TGCTCAATGCCCAAGGGGTAAAT-3′ and H: 5′-AACTCAGATCACGTAGGG-3′) designed by the authors, since those provided by Katsares et al. [35] did not give satisfactory results.

Each 25 µl PCR mixture contained approximately 100 ng of total genomic DNA, 0.32 µM of each primer, 2.5 U of EuroTaq DNA Polymerase (Euroclone), 1× reaction buffer and 200 µM of each dNTP. MgCl_2_ concentration was set at 3 mM, and 12.5 µg of BSA were added to the reaction mixture. PCR amplifications were performed according to the following steps: 1 cycle of 2 min at 94°C, 35 cycles of 1 min at 94°C, 1 min at 46°C and 1 min 30 s at 72°C. A post-treatment of 5 min at 72°C was applied. After electrophoresis on 2% agarose gels, the PCR products were purified using ExoSAP-IT (USB Corporation) and sequenced using an external sequencing core service (Macrogen Europe). Dual peaks of similar height, which could be interpreted as evidence of mitochondrial pseudogenes in the nucleus (Numts) or heteroplasmy, were not observed in any of the electropherograms. The PCR products did not show any occurrence of aspecificity, excluding the possibility of multiple nuclear mtDNA-like sequences.

### Statistical Analysis

Sequences were aligned using the program Clustal W [40] and deposited in GenBank (Accession Nos.: COI, JX854788-JX855023; 16S, JX854562-JX854787). Estimates of the number of polymorphic sites (S), the number of haplotypes (H), haplotype diversity (*h*), nucleotide diversity (*π*), and the mean number of pairwise differences (d) were obtained using the software package DnaSP 5.10 [41].

After having added to our COI and 16S sequences the COI [35], [36] and 16S [35] sequences available in the literature, we constructed two different datasets. One dataset included 236 COI sequences obtained in the present study along with 26 and 49 sequences from the Aegean Sea [35] and Tunisian coasts [36], respectively (see Table 1 for details). The second dataset included the concatenated COI and 16S regions and consisted of the 219 sequences of the present study along with 25 from the Aegean Sea [35]. Concatenating these two mitochondrial genes was appropriate because COI and 16S did not show significant heterogeneity by partition-homogeneity test (*P* = 0.06) performed with PAUP* 4.0b10 [42].

#### a) Analysis of the COI dataset

The presence of population genetic structure was assayed by the Bayesian model-based clustering algorithm implemented in BAPS 5.2 [43]. Clustering was performed using the module for linked molecular data and by applying the codon linkage model, which is appropriate for sequence data. Each analysis was run ten times with a vector of *K*-values = 1 to 22, each with six replicates. Haplotypes were organised into haplogroups following the partition of sequences into the distinct genetic clusters evidenced by Bayesian clustering. The Bayesian clustering algorithms probabilistically assign individuals to groups based on nucleotide frequencies of DNA sequence data without presuming pre-defined populations [44]. For each sampling location, we computed the proportion of a given haplogroup to build maps of haplogroup frequency in the Mediterranean.

Genetic relationships among haplotypes were investigated by a median-joining network [45] using the software package Network 4.5.0.1 (www.fluxus-engineering.com). Mutations were inversely weighted according to the number of times they originated. Therefore, different weights were assigned to the point mutations: sites involved in a single mutational event = 90; sites involved in repeated mutational events = 90/number of repetitions.

Patterns of genetic differentiation at the population level were assessed by Arlequin 3.5.1.3 [46] using a matrix of Tamura and Nei’s [47] genetic distances with a gamma correction according to the best-fitting model of sequence evolution obtained with jModeltest 2.1.1 [48]. First, we calculated pairwise Φ_ST_ values between sampling localities. Samples with less than five individuals were excluded from population level analysis due to a lack of statistical power. We next conducted a hierarchical analysis of molecular variance (AMOVA) [49]. Alternative groupings of populations were taken into account. Initially, we ran analyses in which populations were grouped *a posteriori* according to the pattern of genetic structure suggested by the pairwise Φ_ST_ values. Significance of both pairwise Φ_ST_ values and AMOVA was assessed by a permutation test (with 10,000 random replicates). Then, we defined *a priori* regional groups that corresponded to the main biogeographic subdivisions of the Mediterranean [29]: (1) western vs. eastern Mediterranean; (2) western vs. eastern Mediterranean vs. Adriatic. Sicilian populations located close to the border separating the two Mediterranean basins were twofold tested to be either entirely part of western Mediterranean, or part of western (San Vito lo Capo-SVC, Mondello-MON, Milazzo-MLZ) and eastern (Pachino-PAC, Ognina-OGN) Mediterranean. Where necessary, probability values were corrected for multiple testing by applying the False Discovery Rate method [50].

Aspects of historical demography were inferred by examining the fit of the observed mismatch distribution of DNA pairwise differences to a model of demographic population expansion [51], [52]. Such distributions are unimodal when populations have experienced recent expansion and multimodal at demographic equilibrium or when populations are significantly subdivided. Mismatch distributions and relative Rogers’ parameters [53] (τ, θ_0_, θ_1_) were assessed using Monte-Carlo simulations of 10,000 random samples as implemented in Arlequin 3.5.1.3. The fit of the observed mismatch distribution to the expected distribution under a model of demographic expansion was assessed by Monte-Carlo simulations of 10,000 random samples. The sum of squared deviations (SSD) between observed and expected mismatch distributions was used to test the probability of obtaining a simulated SSD that was larger than or equal to the observed one.

Tajima’s *D* [54] and Fu’s *F*s [55] neutrality tests were used to infer departures from population equilibrium models. Significant negative values of Tajima’s *D* are expected to occur in cases of recent population expansion or after a selective sweep [55]. Positive values are expected in the case of balancing selection, population subdivision or recent bottlenecks [56]. Similarly, significant negative Fu’s *F*s values indicate an excess of rare haplotypes, which might be caused by recent population expansion [55], whereas positive values indicate balancing selection, population structure or moderate bottlenecks [57]. Combining different neutrality tests can help to distinguish among the different evolutionary processes responsible for departures from equilibrium; Fu’s *F*s can better detect demographic expansions, whereas Tajima’s *D* can better detect bottlenecks and populations contractions [58].

#### b) Analysis of the concatenated COI and 16S dataset

Statistical treatments were conducted as with the COI dataset. Note that for this dataset, redefined population groupings were required for some analyses (e.g., AMOVA) due to the lack of 16S sequences from Tunisian coasts.

#### c) Estimates of divergence time

The modal value (τ) of the mismatch distribution of pairwise genetic differences was also used to estimate time since expansion using the formula *t* = τ/2u [51]. Here, *t* is the time since expansion in generations and u is the mutation rate of the entire DNA fragment, equal to u = µs, where µ is the mutation rate per nucleotide per generation, and s is the sequence length. Uncertainty in the estimates of expansion time since expansion was accounted for computing the 95% confidence interval (CI) of *τ* from the parametric bootstrap approach with 10,000 pseudo-replicates [52]. Time since expansion expressed in years was estimated assuming a constant molecular clock, a 2-years generation time [17], and a divergence rate per nucleotide site per million years of 0.52% as estimated in Luttikhuizen et al. [59] for bivalves.

We also used the Bayesian coalescent approach implemented in BEAST 1.7.4 [60] to infer historical demography, as it allows improved recovery of the historical signal within DNA sequences [61]. As there was a lack of 16S sequences for Tunisian populations and of an appropriate divergence rate for 16S region for the species, analysis was carried out on the COI dataset only.

We used the Bayesian skyline plot model [62] assuming a piecewise constant model with ten coalescent intervals; Markov-Chain-Monte-Carlo simulations were run under the GTR+G+I model with four gamma categories using a relaxed uncorrelated molecular clock [62]. Divergence rate of the COI region was modeled using a uniform distribution that ranged from 0.14% to 0.52% (divergence rate per site per million years) according to Luttikhuizen et al. [59]. For each simulation three independent runs were carried out; depending on the size (number of sequences in the dataset) 100 million up to 400 million iterations were used (thinning was adjusted to obtain a final sample of 10,000 records). Tracer 1.5 [63] was used to assess convergence of runs and the Effective Sample Size (ESS) of each parameter. In order to obtain an adequate effective sample size (ESS ≥200), the three independent runs performed for each simulation were combined using the Logcombiner utility of the BEAST 1.7.4 package. The resulting file was used to estimate population size change through time, that was visualized by the Bayesian skyline plot computed with Tracer 1.5.

## Results

### COI Dataset

Specific primers amplified an internal portion of COI 338 base pair (bp) long. Therefore, after the addition to our data of those from Katsares et al. [35] and Rabaoui et al. [36], we obtained a 338 bp sequence alignment from 311 *Pinna nobilis* individuals. Sixty-two different haplotypes (Table S1), defined by 45 polymorphic sites, were found. In our dataset (236 individuals), many haplotypes were found only at a single locality (63%). This percentage increased to 79% when the samples from the Aegean Sea [35] and Tunisian coasts [36] were included. Total mean haplotype and nucleotide diversity, calculated on 311 individuals, were *h* = 0.910 and *π* = 0.007, respectively. The lowest values of haplotype and nucleotide diversity were found among Aegean and Tunisian populations (*h* = 0.720 and *π* = 0.004 for Aegean Sea and *h* = 0.605 and *π* = 0.002 for Tunisian coasts); overall higher values of genetic diversity were found in the other sites. Estimates of COI genetic diversity for each site are reported in Table S2.

#### a) Genetic structuring

**Figure 2:**
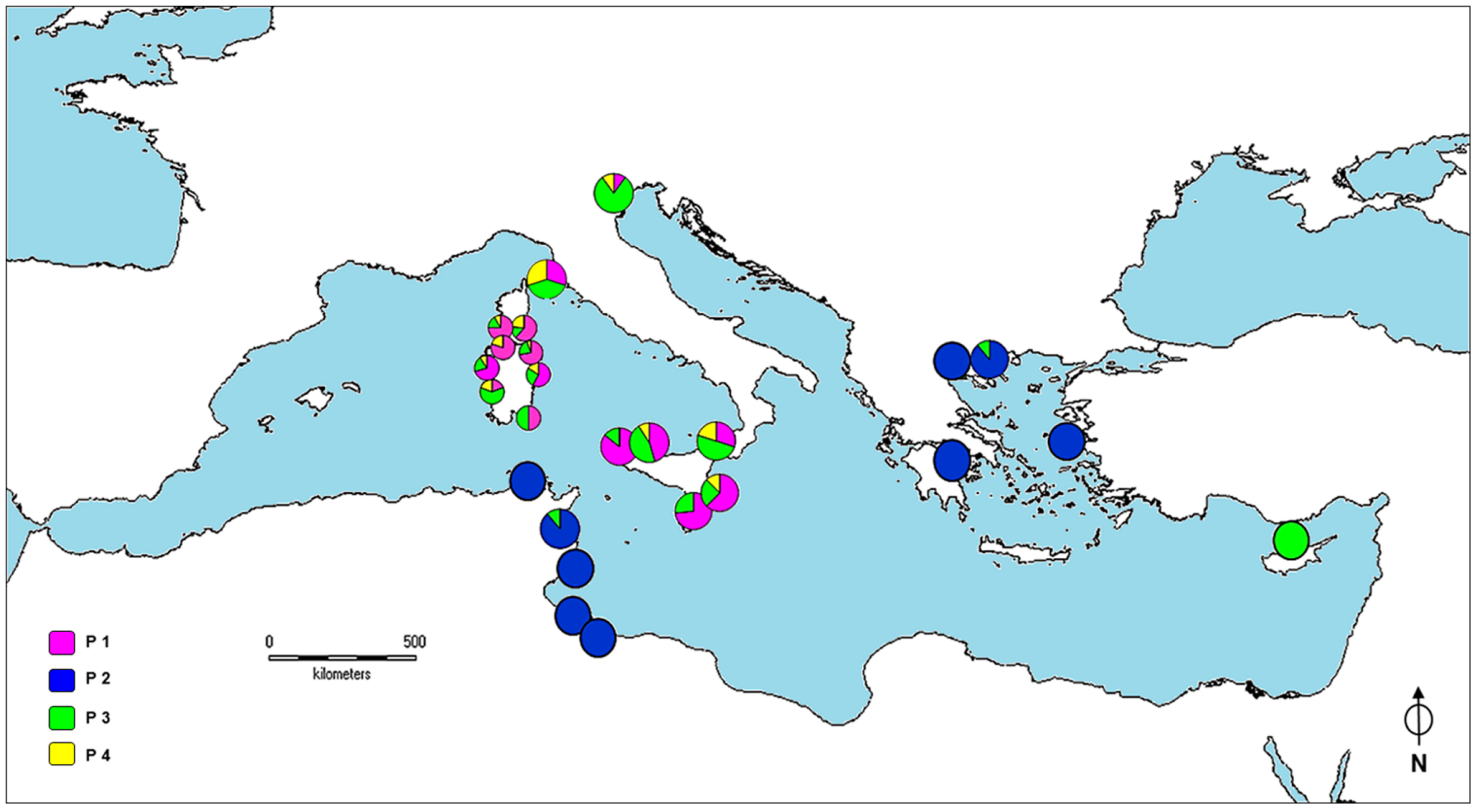
COI dataset: Bayesian cluster distribution. Frequency distribution of the four groups of haplotypes P1, P2, P3, and P4, as evidenced by Bayesian analysis over the Mediterranean map. Due to the high number of populations, the size of the pie charts in the Sardinian-Corsican region was reduced, and the relative populations were merged in three distinct groups as reported below. North-western Sardinia group includes Baia di Porto Conte-BPC, Torre del Porticciolo-POR, Lazzaretto-LAZ, Ospedale Marino-OSM; central-eastern Sardinia group includes Molara-MOL, Capo Ceraso-CCE, Le Saline-SAL, Monte Petrosu-MPE, Porto Ottiolu-OTT; central-western Sardinia group includes Oristano-ORI, Marceddì-MAR, Isola di Mal di Ventre-IMV.

The Bayesian assignment analysis identified four distinct haplotype groups, hereafter denominated P1, P2, P3 and P4 (Table S3). The groups P1, P3 and P4 includeed samples from Sardinia, Corsica, Elba Island, Sicily, the Venetian Lagoon and Cyprus and lack internal geographic structuring, whereas the Aegean and Tunisian samples all exhibited P2 haplotypes, except for one Aegean individual from Epanomi-EP and one Tunisian specimen from Monastir-M, both belonging to group P3. The geographic distributions of haplogroups over the Mediterranean map are provided in Figure 2. The group P1 was the most widespread (41.8%), being present in Sardinia (with the exception of all samples from Isola di Mal di Ventre-IMV), Corsica, Elba Island, Sicily and the Venetian Lagoon (two individuals only). The group P2 was found in 23.5% of individuals and characterised populations from the Aegean Sea and Tunisian coasts. Although P3 was the group spread most widely across the Mediterranean (occurring in all populations except for the majority of the Aegean and Tunisian samples), it occurred at low frequency (26.7%), reaching higher proportion in areas as distant as the northern Adriatic and western Sardinia. The group P4 was characterised by the lowest average frequency (8%) and occurred in north-eastern Sardinia, southern Corsica, at Elba Island, northern Sicily (plus one individual from south-eastern Sicily, Pachino-PAC) and in the Venetian Lagoon (two individuals only).

**Figure 3:**
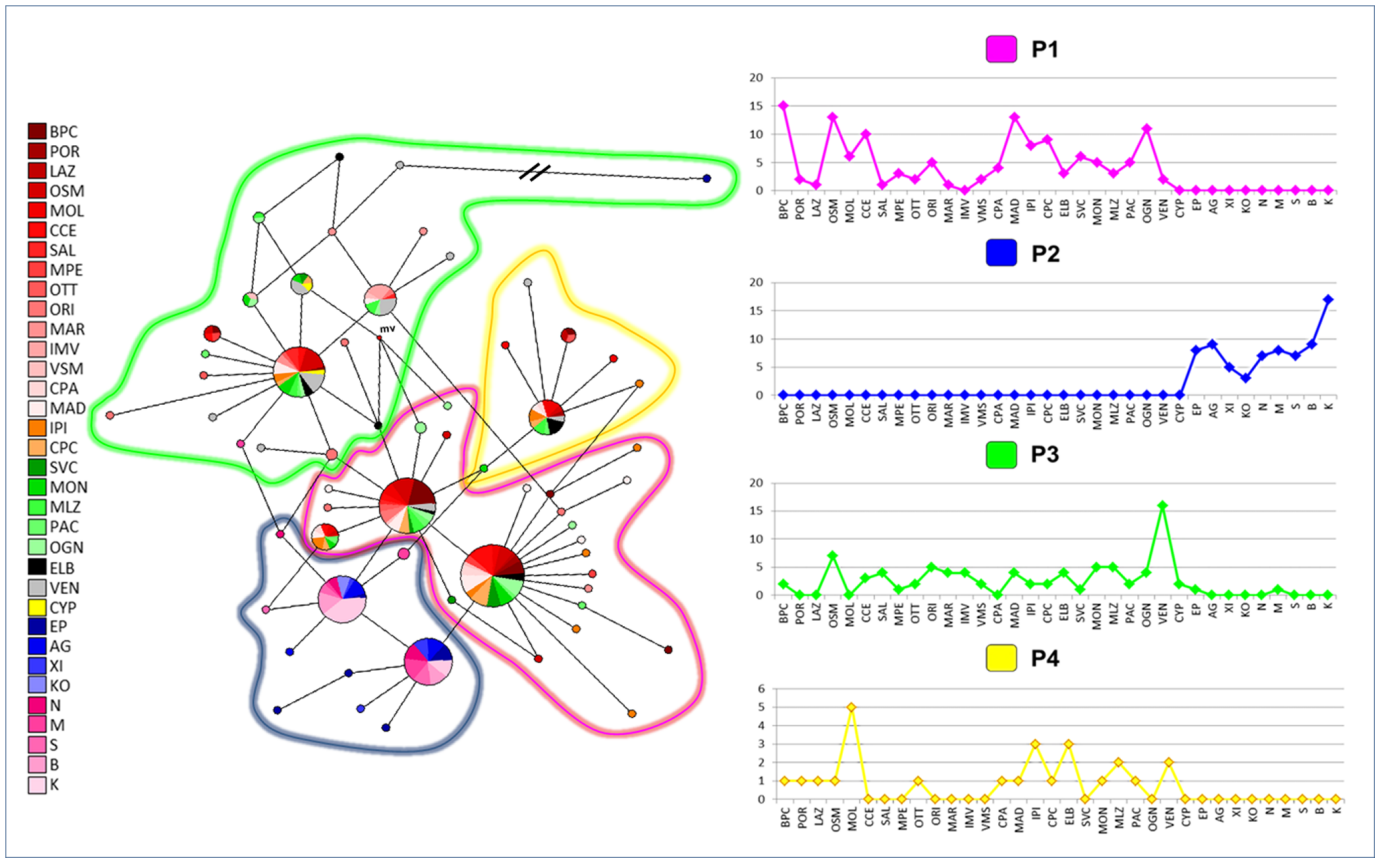
COI dataset: network analysis. Median-joining network (on the left) with haplotypes grouped according to the results of the Bayesian assignment. Small red plots on the nodes, labelled as “mv”, show median vectors representing hypothetic connecting sequences, calculated with a maximum parsimony method. The long branches leading to isolated haplotypes were shortened and indicated with “**\\**”. The distribution of haplogroups within populations (on the right) are displayed in the histogram. The x axis reports populations and the y axis the absolute frequency of distribution. The populations are labelled as reported in Table 1. The number of mutations on the network branches are reported in the Figure S1.

The median-joining network analysis identified a low level of divergence among haplotypes, as, with a few exception, connections were characterised by one or two point mutations, (Figure S1, and Figure 3). Three haplotypes (PN1, PN6, PN7; Table S1), present in 134 of 311 (43.1%) individuals, were the most common among all samples, with the exception of the Aegean and Tunisian samples, of which 85.3% (64 of 75 individuals) showed two main haplotypes (PN52, PN54; Table S1). No evidence of geographic structuring among regions (all characterised by many instances of haplotype sharing) was detected for the entire sample, with the exception of populations from the Aegean Sea and Tunisian coasts that did not share haplotypes with the remaining populations (Figure S1). When haplotypes were grouped on the network according to the results of Bayesian assignment, each cluster (P1, P2, P3 or P4) was generally characterised by one or two widely-dispersed haplotypes, with radiating star-like branching patterns (Figure 3).

The pairwise Φ_ST_ estimates indicated significant genetic differentiation between the Aegean and Tunisian samples and the other sites (Table S4). The Venetian Lagoon was also significantly differentiated in most comparisons, with the only exceptions being three Sardinian populations (Le Saline-SAL, Porto Ottiolu-OTT, Marceddì-MAR) and one Sicilian (Milazzo-MLZ). The AMOVA (Table 2) maximized the largest differences among groups defined *a posteriori* when the samples from the Aegean Sea and Tunisian coasts were grouped together and the samples from Sardinia, Corsica, Elba Island, Sicily and the Venetian Lagoon were considered a unique separate group (Φ_CT_ = 0.369, *P*<0.001) (Table 2, A). However, a similar value of molecular variance (Φ_CT_ = 0.367, *P*<0.001) was obtained when treating the Venetian Lagoon as a separate third group (Table 2, B). When alternative *a priori* groupings of samples were tested, based on biogeographic criteria, the AMOVA showed a decrease in the proportion of Φ_CT_ variance (Table 2, C and D).

**Table 2:** COI dataset: AMOVA.

Source of variation	d.f.	SSD	Var. comp.	% var	Fixation indices	*P*-value
**A - Group 1 (BMC, OSM, MOL, CCE, SAL, OTT, ORI, MAR, CPA, MAD, IPI, CPC, ELB, SVC, MON, MLZ, PAC, OGN, VEN); Group 2 (EP, AG, XI, M, N, S, B, K)**						
Among groups	1	67.784	0.747	36.89	0.369	<0.001
Among populations within groups	24	57.689	0.119	5.90	0.093	<0.001
Within populations	246	284.823	1.158	57.21	0.428	<0.001
**B - Group 1 (BMC, OSM, MOL, CCE, SAL, OTT, ORI, MAR, CPA, MAD, IPI, CPC, ELB, SVC, MON, MLZ, PAC, OGN); Group 2 (EP, AG, XI, M, N, S, B, K); Group 3 (VEN)**						
Among groups	2	85.947	0.703	36.67	0.367	<0.001
Among populations within groups	23	39.526	0.056	2.91	0.046	<0.01
Within populations	246	284.823	1.158	60.41	0.396	<0.001
**C - Group 1, western Mediterranean (BMC, OSM, MOL, CCE, SAL, OTT, ORI, MAR, CPA, MAD, IPI, CPC, ELB, SVC, MON, MLZ); Group 2, eastern Mediterranean (PAC, OGN, VEN, EP, AG, XI, M, N, S, B, K)**						
Among groups	1	19.047	0.116	7.27	0.073	<0.05
Among populations within groups	24	106.427	0.318	19.98	0.215	<0.001
Within populations	246	284.823	1.158	72.75	0.272	<0.001
**D - Group 1, western Mediterranean (BMC, OSM, MOL, CCE, SAL, OTT, ORI, MAR, CPA, MAD, IPI, CPC, ELB, SVC, MON, MLZ); Group 2, eastern Mediterranean, (PAC, OGN, EP, AG, XI,M, N, S, B, K); Group 3, Adriatic Sea (VEN)**						
Among groups	2	54.484	0.353	20.69	0.207	<0.001
Among populations within groups	23	70.990	0.193	11.34	0.143	<0.001
Within populations	246	284.823	1.158	67.96	0.320	<0.001

Results of the analysis of molecular variance (AMOVA). Groups were defined *a posteriori* (A, B) according to the geographic trend emerged from pairwise Φ_ST_ values, or *a priori* (C, D) according to biogeographic criteria. d.f.: degrees of freedom; SSD: sum of squared deviations; var. comp.: variance component; % var: percentage of variation.

#### b) Historical demography

**Figure 4:**
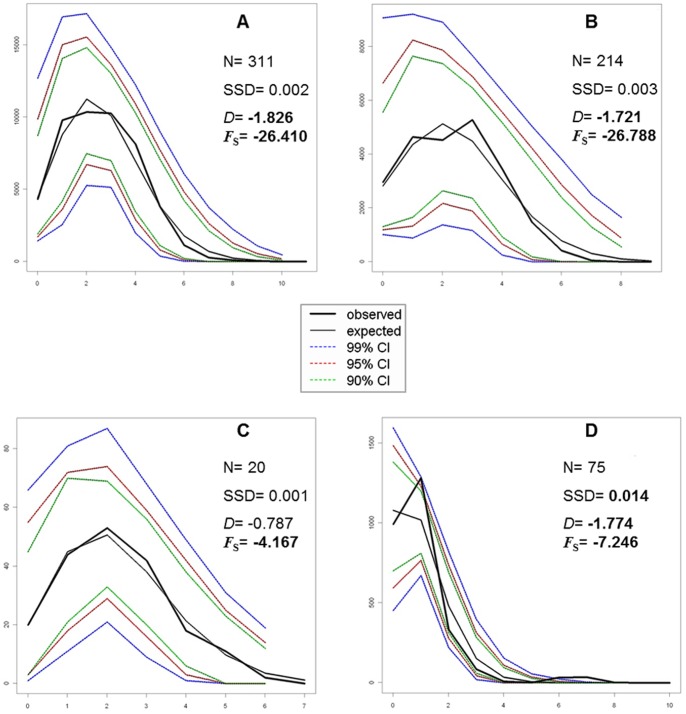
COI dataset: mismatch analysis. Graphs of the mismatch distributions of (A) the entire sample, (B) Sardinia, Corsica, Elba Island, and Sicily, (C) the Venetian Lagoon, and (D) the Aegean Sea and Tunisian coasts. The x axis reports the observed distribution of pairwise nucleotide differences, and the y axis reports the frequencies. N, sample sizes; SSD, sum of squared deviations; *D*, Tajima’s *D* value; *F*s, Fu’s *F* value. The significant values are given in bold.

The mismatch distribution carried out on the entire dataset showed a unimodal distribution of pairwise DNA differences (Figure 4A), fitting the Rogers and Harpending’s model [51] of demographic expansion (SSD = 0.002, *P*>0.05). When assuming the occurrence of discrete groups of populations that, according to AMOVA, represent three geographic areas of (1) Sardinia, Corsica, Elba Island and Sicily, (2) the Venetian Lagoon (Adriatic Sea), and (3) Aegean Sea and Tunisian coasts, we found that the two former groups displayed a unimodal mismatch distribution fitting the demographic expansion model (Figures 4B and 4C), whereas the mismatch distribution of the Aegean and Tunisian samples (Figure 4D) did not correspond to the expected distribution under a demographic expansion model (SSD = 0.014, *P*<0.05). Neutrality tests showed a significant departure from equilibrium, with the exception of Tajima’s *D* for the Venetian Lagoon (Adriatic) population (Figure 4C). The negative values resulting from the neutrality tests were consistent with population expansions in all three groups. The non-significant value of *D* encountered in the Venetian Lagoon may reflect the reduced power of this test compared to the Fu’s *F*s to detect demographic expansions [58].

The time since expansion calculated according to Rogers and Harpending [51] for *P. nobilis* in Mediterranean was estimated to be the Pleistocene. In particular, for populations from Sardinia, Corsica, Elba Island and Sicily, we found that the demographic expansion started around 1.6 Mya (95% CI: 0.512–2.643 Mya). A similar estimate was calculated for the Venetian Lagoon population, with time since expansion dating back to about 1.3 Mya (95% CI: 0.643–1.956 Mya). A more recent demographic expansion was inferred for the Aegean-Tunisian group starting around 0.5 Mya (95% CI: 0.319–0.848 Mya).

**Figure 5:**
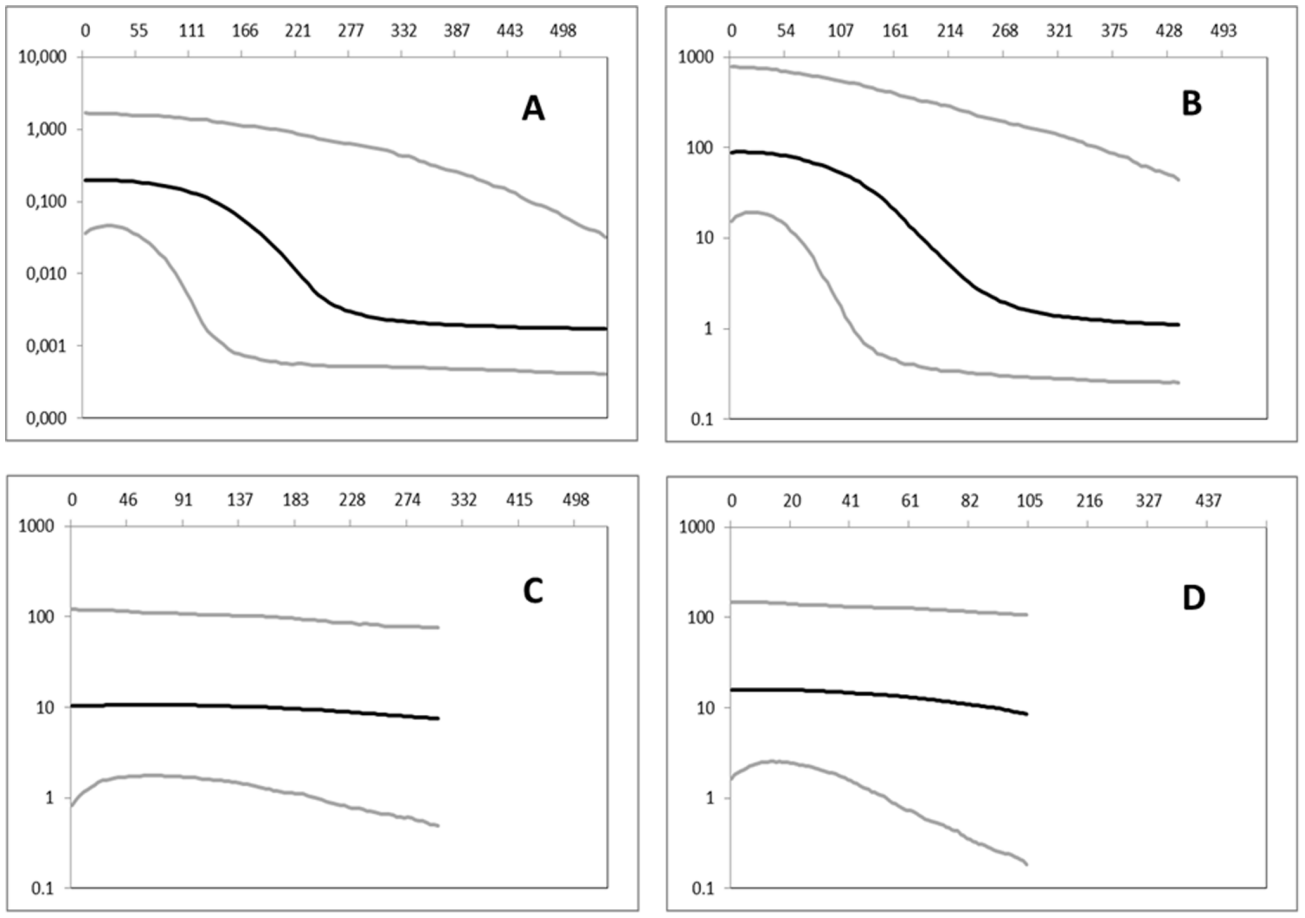
COI dataset: Bayesian skyline plots. Bayesian skyline plots depicting the demographic history of *Pinna nobilis*. The effective population size (y-axis) is shown as a function of time (x-axis), with the units expressed as KYR (1,000 years). (A) entire dataset, (B) the regional group encompassing the Sardinian-Corsican region, Elba Island, and Sicily, (C) the Venetian lagoon and (D) the Aegean Sea and Tunisian coasts. The black lines denote the median population estimates, and the grey lines denote the 95% high posterior density (HPD) confidence limits.

The demographic history inferred by the Bayesian skyline plot revealed a signature of demographic expansion starting approximately 0.270 Mya and ending around 0.100 Mya (Figure 5A). However, only samples from Sardinia, Corsica, Elba Island and Sicily showed a demographic expansion that corresponded to this time interval (Figure 5B). Conversely, the Venetian Lagoon (Figure 5C) and Aegean-Tunisian samples (Figure 5D) had a virtually constant size throughout their demographic history.

### Concatenated COI and 16S Dataset

The concatenated dataset comprised 338 bp for COI and 450 bp for 16S. The sequences of 219 specimens were determined here. Twenty-five COI and 16S sequences from Katsares et al. [35] were added. Among these 244 individuals, 103 haplotypes (Table S5), defined by 78 polymorphic sites (S) were found. Total mean haplotype diversity and nucleotide diversity were *h = *0.961 and *π* = 0.005, respectively. A large number of haplotypes (77%) was found only at a single locality. The Aegean populations showed the lowest average values of haplotype diversity and nucleotide diversity (*h = *0.333 and *π* = 0.001), whereas high levels of genetic diversity were found for almost all other sites. Estimates of genetic diversity for the concatenated COI and 16S dataset are provided in Table S2. Furthermore, estimates of genetic diversity and haplotype frequencies for the 16S region are given in Table S2, and Table S6, respectively.

#### a) Genetic structuring

The Bayesian analysis identified four haplotype groups, hereafter denominated N1, N2, N3 and N4 (Table S7). The most frequent group (N2), representing half of the individuals analysed, included the two most common haplotypes (PNCS 1, PNCS 6; Table S5), found in all populations, with a few exceptions in Sardinia (Isola di Mal di Ventre-IMV), Cyprus and the Aegean Sea (Epanomi-EP, Aggeloxori-AG, and Korinthiakos Gulf-KO). The second group (N3) (32.8% of individuals) included samples from Sardinia, Corsica, Elba Island, Sicily, the Venetian Lagoon and Cyprus. The groups N1 and N4 were less frequent, occurring in 9% and 8.2% of individuals only, respectively. N1 occurred in individuals from Sardinia, Corsica, Elba Island, Sicily and the Venetian Lagoon, while N4 included individuals from all Aegean populations with the exception of those from Xios-XI.

**Figure 6:**
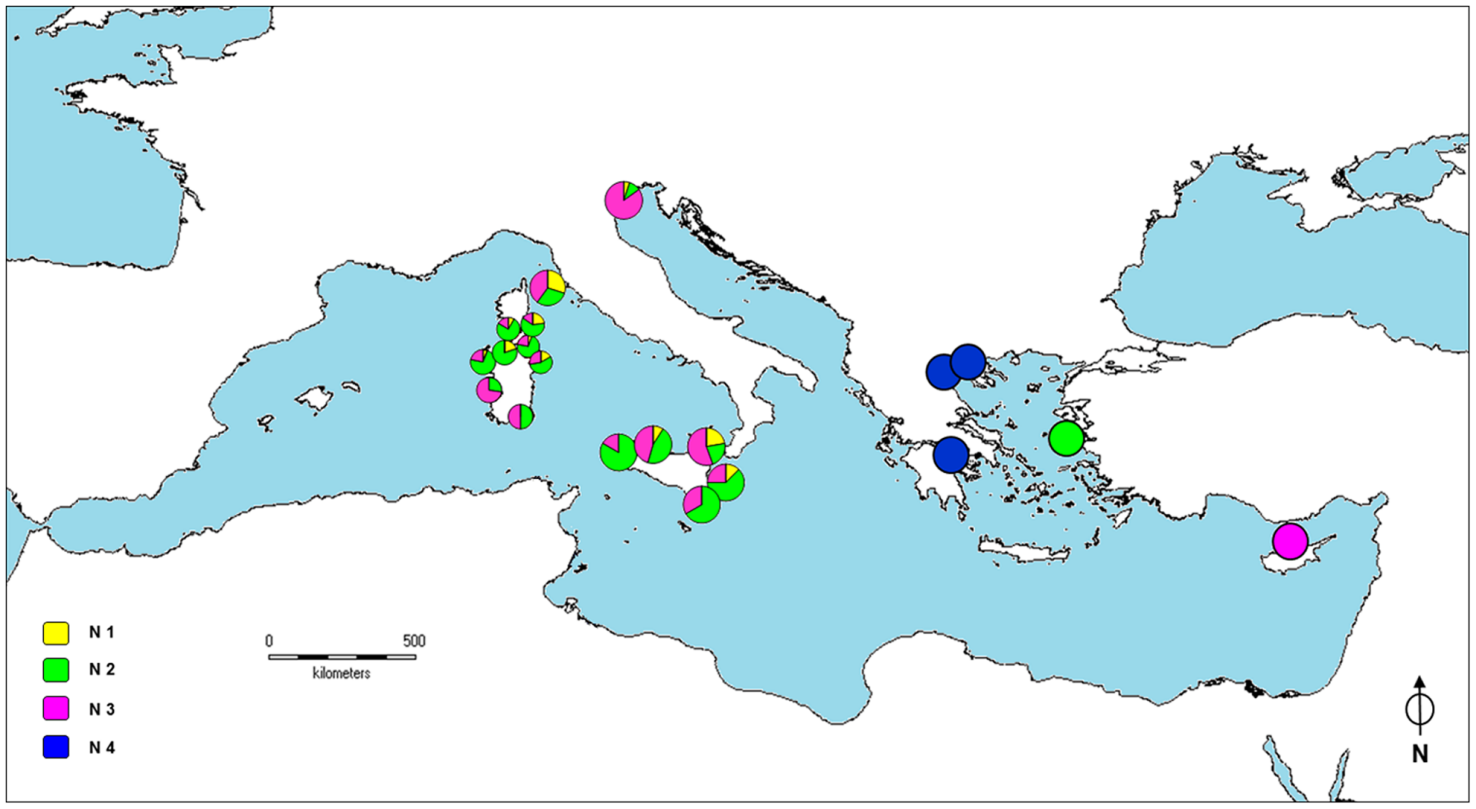
COI-16S dataset: Bayesian cluster distribution. Frequency distribution of the four groups of haplotypes N1, N2, N3 and N4, as evidenced by Bayesian analysis over the Mediterranean map. Due to the high number of populations, the size of the pie charts in the Sardinian-Corsican region was reduced, and the relative populations were merged in three distinct groups as reported below. North-western Sardinia group includes Baia di Porto Conte-BPC, Torre del Porticciolo-POR, Lazzaretto-LAZ, Ospedale Marino-OSM; central-eastern Sardinia group includes Molara-MOL, Capo Ceraso-CCE, Le Saline-SAL, Monte Petrosu-MPE, Porto Ottiolu-OTT; central-western Sardinia group includes Oristano-ORI, Marceddì-MAR, Isola di Mal di Ventre-IMV.

The haplogroups frequency distribution largely resembled that obtained by the COI analysis (Figure 6). In particular, groups N1 and N3 approximately mirrored the distributions of P4 and P3, respectively. A striking exception is represented by haplogroup N2, whose geographic distribution overlapped that of the COI haplogroup P1, spreading as far as the Aegean Sea. At Xios-XI, the N2 group reached frequencies as high as those found in western Mediterranean and Ionian Sea populations. In contrast, the geographic distribution of haplogroup N4 was restricted to the Aegean Sea.

**Figure 7:**
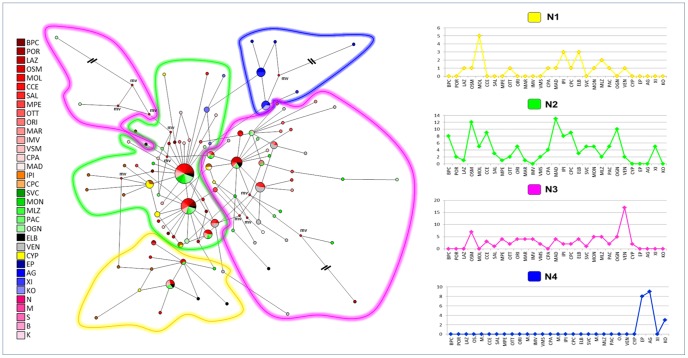
COI-16S dataset: network analysis. Median-joining network (on the left) with haplotypes grouped according to the results of the Bayesian assignment. Small red plots on the nodes, labelled as “mv”, show median vectors, representing hypothetic connecting sequences, calculated with a maximum parsimony method. The long branches leading to isolated haplotypes were shortened and indicated with “**\\**”.The x axis reports populations and the y axis the absolute frequency of distribution. The populations are labelled as reported in Table 1. The number of mutations on the network branches are reported in the Figure S2.

The median-joining network of haplotypes (Figure S2 and Figure 7) showed a pattern of high genetic variability distributed across the Mediterranean. The two most frequent haplotypes (PNCS 1, PNCS 6; Table S5) were found in 23.8% of individuals and were shared only among individuals from Sardinia, Elba Island and Sicily. Different star-like branching patterns radiating out from the inferred root haplotypes occurred in the network (Figure S2). A few point mutations (1 to 6) distinguished haplotypes from each other, and a diffused unresolved reticulation of branches was found among individuals. Haplotypes belonging to the Venetian Lagoon and Aegean Sea populations occupied a peripheral position in the network. The two Cyprian haplotypes were very divergent from one another (Figure S2). No evidence of spatial structuring was apparent at either the local or large geographic scales. However, an exception was represented by Aegean individuals, which did not share haplotypes with other samples. Considering groupings detected by Bayesian assignment analysis (Figure 7), the network showed that the most diffused group (N2) was characterised by two main haplotypes and many derived ones, that diverged for a single point mutation. The group N3 was also characterised by a high level of haplotype variation, with unique haplotypes radiating from the central ones. The group N1 exhibited a similar pattern, although a reduced level of polymorphism was found. The group N4 showed the lowest level of diversity.

The pairwise Φ_ST_ estimates obtained for the concatenated dataset (Table S8) were similar to those reported for COI. A significant genetic differentiation occurred between the Aegean samples of Epanomi-EP and Aggeloyesori-AG, and the other samples. As previously noticed, the Venetian Lagoon sample was significantly differentiated from the others in most comparisons with the only exceptions being two Sardinian populations (Le Saline-SAL, Porto Ottiolu-OTT). Nonetheless, combining data from different genes resulted in a larger number of significant comparisons (Table S8). In particular, samples from Corsica appeared to be genetically differentiated not only from samples outside the Sardinian-Corsican region, but also from their northern Sardinian counterparts, and samples from Xios-XI were significantly different from those from the Aegean Sea.

Consistently with results of the COI analysis, AMOVA (Table 3) detected maximum genetic differentiation among groups defined *a posteriori* on the pairwise Φ_ST_ values, when samples from the Aegean Sea were grouped separately from the others (Φ_CT_ = 0.396, *P*<0.001) (Table 3, A). A similarly high level of molecular variance was found when the Venetian Lagoon was considered as a third group (Φ_CT_ = 0.356, *P*<0.001) (Table 3, B). AMOVA tested on *a priori* groupings of samples also showed a decrease in the proportion of variance (Table 3, C and D); particularly no significant differences were found between groups corresponding to the western and eastern Mediterranean (Φ_CT_ = 0.045, *P*>0.05) (Table 3, C and D).

**Table 3:** COI-16S dataset: AMOVA.

Source of variation	d.f.	SSD	Var. comp.	% var	Fixation indices	*P*-value
**A - Group 1 (BMC, OSM, MOL, CCE, SAL, OTT, ORI, MAR, CPA, MAD, IPI, CPC, ELB, SVC, MON, MLZ, PAC, OGN, VEN); Group 2 (EP, AG, XI)**						
Among groups	1	52.953	1.221	39.61	0.396	<0.001
Among populations within groups	20	104.776	0.370	12.00	0.199	<0.001
Within populations	201	299.754	1.491	48.38	0.516	<0.001
**B - Group 1 (BMC, OSM, MOL, CCE, SAL, OTT, ORI, MAR, CPA, MAD, IPI, CPC, SVC, MON, MLZ, PAC, OGN, ELB); Group 2 (EP, AG, XI); Group 3 (VEN)**						
Among groups	2	80.326	0.971	35.56	0.356	<0.001
Among populations within groups	19	77.403	0.268	9.80	0.152	<0.001
Within populations	201	299.754	1.491	54.64	0.454	<0.001
**C - Group 1,western Mediterranean (BMC, OSM, MOL, CCE, SAL, OTT, ORI, MAR, CPA, MAD, IPI, CPC, SVC, MON, MLZ, ELB); Group 2, eastern Mediterranean (PAC, OGN, VEN, EP, AG, XI)**						
Among groups	1	17.491	0.096	4.48	0.045	>0.05
Among populations within groups	20	140.238	0.558	26	0.272	<0.001
Within populations	201	299.754	1.491	69.62	0.305	<0.001
**D - Group 1, western Mediterranean (BMC, OSM, MOL, CCE, SAL, OTT, ORI, MAR, CPA, MAD, IPI, CPC, SVC, MON, MLZ, ELB); Group 2, eastern Mediterranean, (PAC, OGN, EP, AG, XI); Group 3, Adriatic Sea (VEN)**						
Among groups	1	53.206	0.376	16.44	0.164	<0.01
Among populations within groups	19	104.524	0.421	18.39	0.220	<0.001
Within populations	201	299.574	1.491	65.17	0.348	<0.001

Results of the analysis of molecular variance (AMOVA). Groups were defined *a posteriori* (A, B) according to the geographic trend emerged from pairwise Φ_ST_ values, or *a priori* (C, D) according to biogeographic criteria. d.f.: degrees of freedom; SSD: sum of squared deviations; var. comp.: variance component; % var: percentage of variation.

#### b) Historical demography

**Figure 8:**
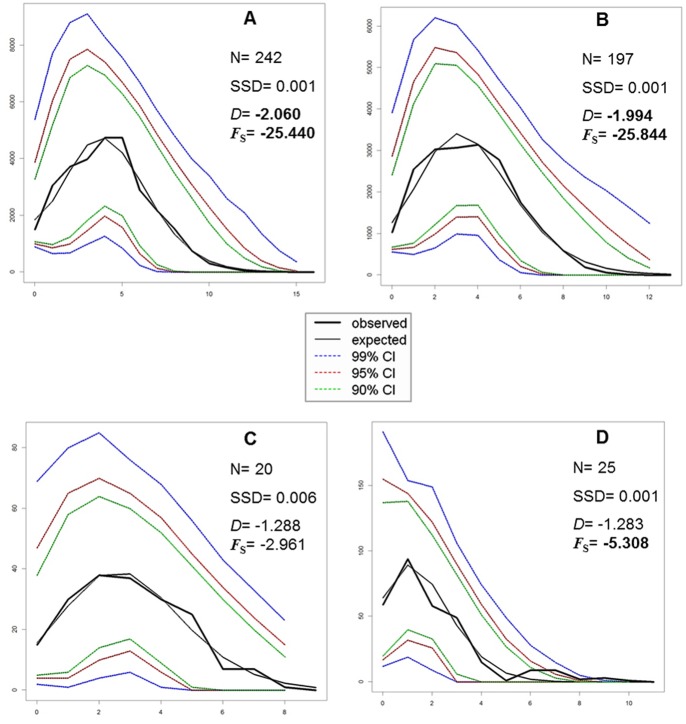
COI-16S dataset: mismatch analysis. Graphs of the mismatch distributions of (A) the entire sample, (B) Sardinia, Corsica, Elba Island, and Sicily, (C) the Venetian Lagoon and (D) the Aegean Sea. The x axis reports the observed distribution of pairwise nucleotide differences, and the y axis reports the frequencies. N, sample sizes; SSD, sum of squared deviations; *D*, Tajima’s *D* value; *F*s, Fu’s *F* value. The significant values are given in bold.

The results of demographic analyses of the concatenated dataset partly agreed with those of the COI dataset. The mismatch distribution carried out on the entire dataset fitted the model of demographic expansion (SSD = 0.001, *P*>0.05), with a unimodal distribution of pairwise DNA differences (Figure 8A). In contrast to the results obtained for the COI dataset, all the three groups, (1) Sardinia, Corsica, Elba Island and Sicily (Figure 8B); (2) the Venetian Lagoon (Figure 8C); and (3) the Aegean Sea (Figure 8D), displayed unimodal mismatch distributions fitting the model of demographic expansion. Nonetheless, only the group including samples from Sardinia, Corsica, Elba Island and Sicily (Figure 8B) showed significant negative values for Tajima’s *D* and Fu’s *F*s, which can be consistent with a population expansion model. The non-significant values of neutrality tests obtained for the populations from the Venetian Lagoon and the Aegean Sea (Figure 8C, 8D) were likely to reflect the reduced power of the tests to detect departures from equilibrium, due to small sample size. Indeed, when we used only samples from Aegean Sea, Fu's *F*s statistic did not displayed a significant departure from equilibrium for the COI dataset too (data not shown).

## Discussion

This study provides the first analysis on mtDNA genetic variation of *Pinna nobilis* populations from a wide study area, roughly corresponding to the central part of the western Mediterranean, Ionian Sea and Adriatic Sea ecoregions (*sensu* Spalding et al. [30]). Supplementing the sequences obtained in the present study with those from previous investigations conducted in the Aegean Sea and along the Tunisian coasts provided a deep insight into (1) the large-scale patterns of spatial genetic variation of *P. nobilis* and (2) the role of Mediterranean biogeographic boundaries in shaping the species’ genetic structuring.

The high variability of the mtDNA COI region of *P. nobilis* further supports the effectiveness of this marker in investigating the genetic architecture of marine species (see [64] and references therein). In addition, the sample sizes adopted in the present survey allowed us to identify 16S haplotypes undetected in the previous study by Katsares et al. [35]. As the results obtained from the analysis of the COI sequences and of the merged COI and 16S sequences were largely consistent, we discuss them jointly in the following sections.

### Genetic Structure of *Pinna nobilis* in the Mediterranean

Our study showed the occurrence of a moderate level of genetic structuring in *P. nobilis* populations in the study area. The low genetic divergence among the haplogroups and haplotypes, as evinced by the reduced number of point mutations separating the sequences from each other, suggested a common origin of the *P. nobilis* populations. However, Bayesian assignment analysis, pairwise Φ_ST_ values and AMOVA noted the occurrence of at least two main genetically divergent groups of populations: (1) the Sardinian-Corsican region, Elba Island and Sicily (western Mediterranean and Ionian Sea) and (2) the Aegean Sea and Tunisian coasts (eastern Mediterranean).

We detected a primary, significant pattern of genetic structuring along the West-East direction, with samples from the Aegean Sea (excluding the Xios-XI sample for the concatenated COI and 16S dataset) and Tunisian coasts, which were genetically differentiated from the remaining samples. This pattern could be explained as a possible consequence of a reduced gene flow between the western and eastern Mediterranean. Several studies have shown that a hindrance to gene flow exists between the western and eastern Mediterranean, even for species with high dispersal capabilities and large population sizes (e.g., [65], [66], [67]). This result was partially consistent with findings reported for other bivalves with a high potential for dispersal, along with significant genetic differentiation and a pattern of isolation by distance (e.g., [68], [69], [70]).

Other studies have also shown that the genetic break between the western and eastern Mediterranean corresponds to the Siculo-Tunisian Strait (e.g., [71]); nonetheless, for different marine organisms the geographic position of the genetic break may shift eastward with respect to the strait (e.g., [29], [72] and references therein). In our case, the pattern of genetic structuring between the Aegean with Tunisian samples and other populations (Sardinian-Corsican region, Elba Island and Sicily) did not mirror the West-East separation of the Mediterranean along the Siculo-Tunisian Strait (see [73]). Moreover, the sample from the southern coast of the strait (Tunisian populations), which was homogeneous with the Aegean populations, was genetically divergent from the samples of the northern coast of the strait (Ionian Sicily), suggesting the occurrence of a further genetic break in a North-South direction. This finding could be due to the hydrodynamic characteristics of this area: the Algerian Current, which flows eastward from the Strait of Gibraltar, along the northern coasts of Africa and passes over the Siculo-Tunisian Strait [39], represents a hydrodynamic barrier between the facing coasts of Tunisia and Sicily. Such a barrier, which reduces the connectivity among populations via marine currents, has also been invoked for the identification of distinct mitochondrial lineages in the vermetid gastropod *Dendropoma petraeum* in adjacent locations in Tunisia and Sicily [74]. Furthermore, the genetic affinity of the Sicilian samples with the western Mediterranean ones, led us to hypothesise a shift of the boundary of the genetic break eastward from the Siculo-Tunisian Strait, as proposed by Pérès and Picard [75] and Bianchi [29]. Although few studies on population genetics involve this area, a similar picture has been reported for *Patella rustica* [76], whereby the western mtDNA lineage reaches as far as the southern coast of the Italian Peninsula, well beyond the Siculo-Tunisian Strait. A genetic break located at the level of the southern tip of Calabria has also been described for *Posidonia oceanica* [77]. However, at this stage of knowledge, we cannot rule out that the genetic pattern observed around the western-eastern edge of the Mediterranean may reflect a primary genetic break in a North-South direction along the Siculo-Tunisian Strait. Furthermore, as has already been reported for other species [77], the genetic homogeneity found between the samples from the Tunisian coasts and the Aegean Sea could be ascribed to the persistence of a relict population in the eastern, semi-enclosed basin, rather than to an effective gene flow between the two Mediterranean basins. More individuals from unsampled regions of both western and eastern Mediterranean are needed to disentangle this point.

Our results also suggest the occurrence of a weak but significant genetic divergence of the Venetian Lagoon population from the above-mentioned two groups of populations. This finding is further evidence of the semi-enclosed nature of the Adriatic Sea, which represents a well-defined phylogeographic region within the Mediterranean [13]. A genetic discontinuity between the Adriatic Sea and the rest of Mediterranean has also been found in other species, such as the common cuttlefish *Sepia officinalis* [78], and the sea urchin *Paracentrotus lividus* [79]. Furthermore, several studies have revealed the occurrence of a genetic break among the Adriatic and Aegean seas, that was explained by the hydrographical isolation of these two basins [80], [81], [82], [83].

Interestingly, samples from Cyprus, at the easternmost part of the Mediterranean, exhibited haplotypes typical of the westernmost populations. The human-mediated introduction of planktonic larvae passively transported in the ballast water of commercial ships is a common finding in marine molluscs invasion [84], [85], [86], [87] and may account for this outcome. However, the small number of samples from Cyprus prevented any statistically supported inference.

### Historical Demography

The results of historical demographic analysis and the estimates of time since expansion lead to the hypothesis of a Pleistocene scenario for *P. nobilis*, with expansions/contractions of its populations due to sea-level fluctuations of this period, repeatedly leading to the isolation, or partial isolation, of the Mediterranean basins [88]. Conversely, vicariant events, leading to ancient divergence of populations, are unlikely to have occurred in *P. nobilis* because we would expect a bimodal mismatch distribution of pairwise DNA differences for the entire dataset, which may occur when different genetic lineages are present [11], [89]. It should be noted that the estimates of time since expansion may be biased by the lack of a mutation rate specific for *P. nobilis* and the time dependency of molecular rate estimates [90]. In the present investigation, we used a range of inter-specific divergence rates estimated for bivalves [59]. However, the estimated molecular rates were much higher over short timescales at the intra-specific level than between species ([90], [91] and references therein but see also [92]). Despite these caveats, our results, nonetheless, provide initial insights into the dynamics of the colonisation of *P. nobilis* populations in the Mediterranean.

All populations analysed in this study are likely to have shared a common origin. Our data suggest an eastward expansion across the Mediterranean, likely mediated by marine currents (e.g., the Algerian Current), and followed by one or more founder events, that lead to lower diversity and private haplotypes in the Aegean and Tunisian populations. A similar trend was also reported for populations of the sea cucumber *Holothuria mammata* from the Aegean Sea [65]. In particular, the group of populations including the Sardinian-Corsican region, Elba Island, Sicily and the Venetian Lagoon samples survived the Late Pleistocene climatic cycles. Our data also suggest that the Sardinian-Corsican region, Elba Island and Sicily populations further experienced their last demographic expansion during the Pleistocene glaciation. Conversely, no evidence of demographic expansion was detected for the Venetian Lagoon, Aegean and Tunisian populations, with the exception of a weak trace of expansion that may have occurred later in the Early-Middle Pleistocene.

### Final Remarks

Rabaoui et al. [36] suggested that the conservation and management of *P. nobilis* should benefit from improvements in the sampling plan (with the inclusion of samples from the western Mediterranean). Although part of the range of the species remains unsampled, our study represents an effort in this direction, as it triples the existing genetic information on *P. nobilis* with new data from a wide area of the western Mediterranean.

The general picture revealed by our study is the presence of a large, homogeneous group of populations of *P. nobilis* spanning two Mediterranean marine ecoregions (the western Mediterranean and Ionian Sea), which genetically diverges from the Adriatic population and those from the Aegean Sea and Tunisian coasts. The semi-enclosed nature of the Adriatic Sea may explain the genetic divergence of the Venetian Lagoon population. Hindrances to gene flow, that are related to biogeographic boundaries among these Mediterranean sectors, and the Pleistocene changes in sea levels, can be invoked to explain the pattern of genetic structuring shown in this study. Our results further suggest the occurrence of a West-East genetic break located eastward of the Siculo-Tunisian Strait, and also provide evidence of a further genetic break in this area which is in the North-South direction. In this context, it would be worth noting that other samples from the western (e.g. Spain and Balearic Islands) and eastern Mediterranean (e.g. from Egypt and Israel) would be very informative for the understanding of the species’ evolutionary history.

Interestingly, although the data for the local or regional levels of genetic erosion due either to human disturbance or to bio-ecological changes are unavailable, the higher levels of mitochondrial variability found in the Sardinian-Corsican region, Elba Island, Sicily and the Venetian Lagoon relative to the populations from the Aegean Sea and Tunisian coasts support the occurrence of an eastward demographic expansion. The lack of strong genetic divergence among these three geographic areas (e.g., only one point mutation separates the Aegean and Tunisian samples from those from Sardinia, Corsica, Elba Island, Sicily and the Venetian Lagoon) is evidence of a common origin of *P. nobilis* populations in the Mediterranean.

From a conservation point of view, the three genetically divergent groups, (1) the samples from Sardinia, Corsica, Elba Island and Sicily, (2) the samples from the Aegean Sea and Tunisian coasts and (3) the Venetian Lagoon sample, should be considered as different management units. Moreover, the samples from the Venetian Lagoon may be considered a “peripheral isolate” *sensu* Frey [93], with particular conservation relevance, given its peculiar geographic position.

## Supporting Information

Figure S1 **COI dataset: network analysis.** Median-joining network showing the haplotypes relationships among *Pinna nobilis* populations. Small red plots on the nodes, labelled as “mv”, show median vectors, representing hypothetic connecting sequences, calculated with a maximum parsimony method. Haplotypes diverge each other for a single mutation except where Arabic numbers on network branches indicate the occurrence of a higher number of point mutations. Populations are labelled as reported in Table 1.
(TIF)

Figure S2 **COI-16S dataset: network analysis.** Median-joining network showing the haplotypes relationships among *Pinna nobilis* populations. Small red plots on the nodes, labelled as “mv”, show median vectors, representing hypothetic connecting sequences, calculated with a maximum parsimony method. Haplotypes diverge each other for a single mutation except where Arabic numbers on network branches indicate the occurrence of a higher number of point mutations. Populations are labelled as reported in Table 1.
(TIF)

Table S1 **COI dataset: haplotype frequencies.** Frequency distribution of COI haplotypes in 311 individuals from 34 populations of *Pinna nobilis*. N: absolute frequency; %: relative frequency within Mediterranean populations. Populations are labelled as in Table 1.
(DOC)

Table S2 **COI, COI-16S, 16S datasets: genetic divergence estimates.** Sample sizes and genetic diversity estimates obtained for the mitochondrial regions analysed in *Pinna nobilis*. N: sample sizes; S: number of polymorphic sites; H: number of haplotypes; *h*: haplotype diversity; *π*: nucleotide diversity; d: mean of pairwise nucleotide differences. Populations are labelled as in Table 1. Sites with gaps were not considered.
(DOC)

Table S3 **COI dataset: Bayesian COI haplogroup frequencies.** N: absolute frequency; %: relative frequency within Mediterranean populations of *Pinna nobilis*. Populations are labelled as in Table 1.
(DOC)

Table S4 **COI dataset: pairwise Φ_ST_ values among sampling localities.** Pairwise Φ_ST_ values between *Pinna nobilis* populations with at least five individuals. Significance was assessed by permutation test. Significant values after correction for multiple testing are reported in bold. Populations are labelled as in Table 1.
(DOC)

Table S5 **COI-16S dataset: haplotype frequencies.** Frequency distribution of mitochondrial haplotypes in 244 individuals from 29 populations of *Pinna nobilis*. N: absolute frequency; %: relative frequency within Mediterranean populations. Populations are labelled as in Table 1.
(DOC)

Table S6 **16S dataset: haplotype frequencies.** Frequency distribution of haplotypes in 251 individuals from 29 populations of *Pinna nobilis*. N: absolute frequency; %: relative frequency within Mediterranean populations. Populations are labelled as in Table 1.
(DOC)

Table S7 **COI-16S dataset: Bayesian COI-16S mitochondrial region haplogroup frequencies.** N: absolute frequency; %: relative frequency within Mediterranean populations of *Pinna nobilis*. Populations are labelled as in Table 1.
(DOC)

Table S8 **COI-16S dataset: pairwise Φ_ST_ values among samples localities.** Pairwise Φ_ST_ values between *Pinna nobilis* populations with at least five individuals. Significance was assessed by permutation test. Significant values after correction for multiple testing are reported in bold. Populations are labelled as in Table 1.
(DOC)
