# Supplementary figures and images for: Mitochondrial DNA Reveals Genetic Structuring of Pinna nobilis across the Mediterranean Sea
Source: PLoS One. 2013 Jun 28;8(6):e67372. doi: 10.1371/journal.pone.0067372 (PMC3696058; doi:10.1371/journal.pone.0067372)

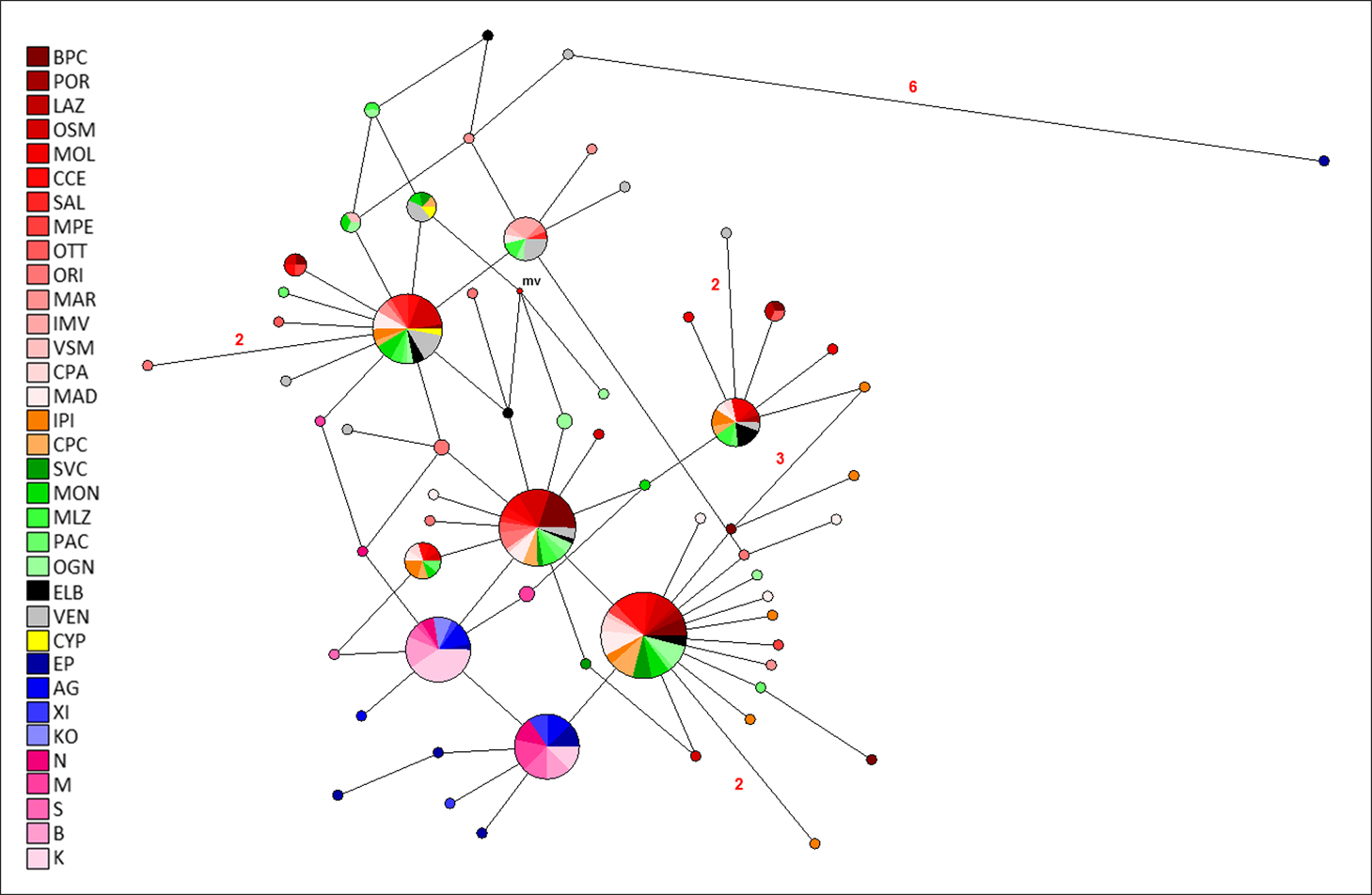

Supplement: Figure S1 — COI dataset: network analysis. Median-joining network showing the haplotypes relationships among Pinna nobilis populations. Small red plots on the nodes, labelled as “mv”, show median vectors, representing hypothetic connecting sequences, calculated with a maximum parsimony method. Haplotypes diverge each other for a single mutation except where Arabic numbers on network branches indicate the occurrence of a higher number of point mutations. Populations are labelled as reported in Table 1. (TIF) [file pone.0067372.s001.tif]

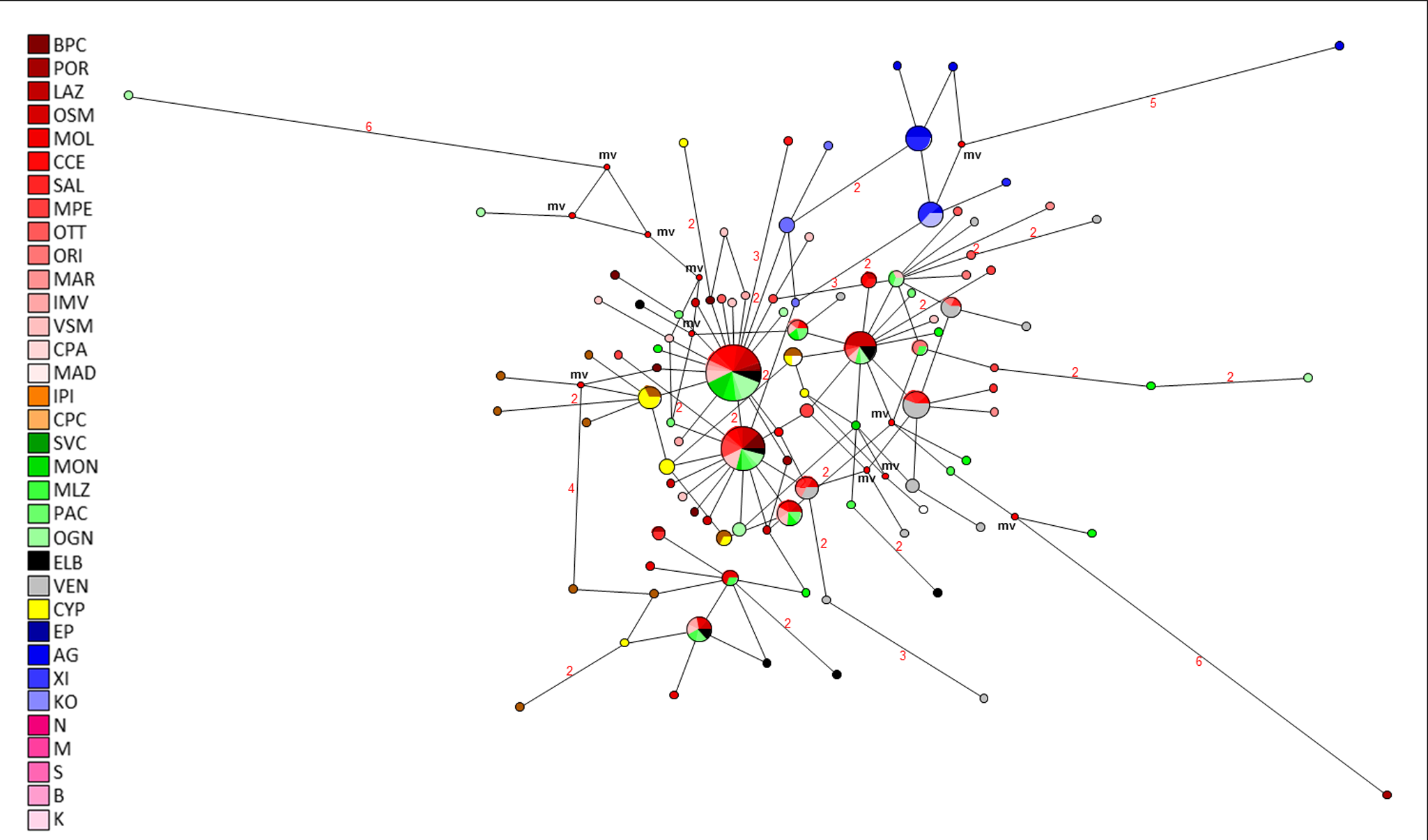

Supplement: Figure S2 — COI-16S dataset: network analysis. Median-joining network showing the haplotypes relationships among Pinna nobilis populations. Small red plots on the nodes, labelled as “mv”, show median vectors, representing hypothetic connecting sequences, calculated with a maximum parsimony method. Haplotypes diverge each other for a single mutation except where Arabic numbers on network branches indicate the occurrence of a higher number of point mutations. Populations are labelled as reported in Table 1. (TIF) [file pone.0067372.s002.tif]
