# Supplementary material for: Mitochondrial DNA Reveals Genetic Structuring of Pinna nobilis across the Mediterranean Sea
Source: PLoS One. 2013 Jun 28;8(6):e67372. doi: 10.1371/journal.pone.0067372 (PMC3696058; doi:10.1371/journal.pone.0067372)
Supplement: Table S1 — COI dataset: haplotype frequencies. Frequency distribution of COI haplotypes in 311 individuals from 34 populations of Pinna nobilis. N: absolute frequency; %: relative frequency within Mediterranean populations. Populations are labelled as in Table 1. (DOC) [file pone.0067372.s003.doc]

| **Clade** | **N** | **%** | **Populations** | **# GenBank** |
| --- | --- | --- | --- | --- |
| PN 1 | 44 | 14.15 | BMC-OSM-MOL-CCE-SAL-OTT-ORI-VSM-MAD-CPC- ELB- SVC-MLZ-PAC-OGN- VEN | JX854788 |
| PN 2 | 1 | 0.32 | BMC | JX854791 |
| PN 3 | 1 | 0.32 | BMC | JX854795 |
| PN 4 | 3 | 0.96 | BMC-LAZ-OTT | JX854797 |
| PN 5 | 4 | 1.29 | BMC-OSM-CCE-MPE | JX854798 |
| PN 6 | 54 | 17.36 | BMC-POR-LAZ-OSM-MOL-CCE-MPE-VSM-CPA-MAD-IPI-CPC-ELB-SVC-MON-PAC-OGN | JX854799 |
| PN 7 | 36 | 11.58 | BMC-OSM-CCE-SAL-OTT-MAR-MAD-IPI-CPC-ELB-MON-MLZ-PAC-OGN-VEN-CYP | JX854800 |
| PN 8 | 17 | 5.47 | POR-OSM-MOL-CPA-MAD-IPI-CPC-ELB-MLZ-PAC-VEN | JX854808 |
| PN 9 | 1 | 0.32 | OSM | JX854814 |
| PN 10 | 1 | 0.32 | OSM | JX854817 |
| PN 11 | 10 | 3.22 | OSM-MOL-CCE-CPA-MAD-IPI-CPC-MON-PAC | JX854824 |
| PN 12 | 1 | 0.32 | MOL | JX854835 |
| PN 13 | 1 | 0.32 | MOL | JX854841 |
| PN 14 | 15 | 4.82 | SAL-ORI-IMV-VSM-MAD-MLZ-OGN-VEN | JX854857 |
| PN 15 | 1 | 0.32 | MPE | JX854861 |
| PN 16 | 1 | 0.32 | OTT | JX854865 |
| PN 17 | 1 | 0.32 | ORI | JX854870 |
| PN 18 | 1 | 0.32 | ORI | JX854873 |
| PN 19 | 2 | 0.64 | ORI | JX854874 |
| PN 20 | 1 | 0.32 | ORI | JX854877 |
| PN 21 | 1 | 0.32 | ORI | JX854878 |
| PN 22 | 1 | 0.32 | MAR | JX854879 |
| PN 23 | 1 | 0.32 | MAR | JX854880 |
| PN 24 | 1 | 0.32 | MAR | JX854881 |
| PN 25 | 3 | 0.96 | VSM-MON-OGN | JX854891 |
| PN 26 | 1 | 0.32 | MAD | JX854903 |
| PN 27 | 1 | 0.32 | MAD | JX854905 |
| PN 28 | 1 | 0.32 | MAD | JX854907 |
| PN 29 | 1 | 0.32 | MAD | JX854908 |
| PN 30 | 1 | 0.32 | IPI | JX854916 |
| PN 31 | 1 | 0.32 | IPI | JX854920 |
| PN 32 | 1 | 0.32 | IPI | JX854922 |
| PN 33 | 1 | 0.32 | IPI | JX854923 |
| PN 34 | 1 | 0.32 | IPI | JX854924 |
| PN 35 | 7 | 2.25 | CPC-SVC-MON-VEN-CYP | JX854929 |
| PN 36 | 1 | 0.32 | SVC | JX854947 |
| PN 37 | 1 | 0.32 | MON | JX854957 |
| PN 38 | 2 | 0.64 | MLZ-OGN | JX854964 |
| PN 39 | 1 | 0.32 | PAC | JX854973 |
| PN 40 | 1 | 0.32 | PAC | JX854974 |
| PN 41 | 1 | 0.32 | OGN | JX854977 |
| PN 42 | 1 | 0.32 | OGN | JX854986 |
| PN 43 | 2 | 0.64 | OGN | JX854987 |
| PN 44 | 1 | 0.32 | ELB | JX854998 |
| PN 45 | 1 | 0.32 | ELB | JX855000 |
| PN 46 | 1 | 0.32 | VEN | JX855003 |
| PN 47 | 1 | 0.32 | VEN | JX855005 |
| PN 48 | 1 | 0.32 | VEN | JX855010 |
| PN 49 | 1 | 0.32 | VEN | JX855012 |
| PN 50 | 1 | 0.32 | VEN | JX855016 |
| PN 51 | 1 | 0.32 | EP | DQ448216 |
| PN 52 | 32 | 10.29 | EP-AG- XI-N-M-S-B-K | DQ448217 |
| PN 53 | 1 | 0.32 | EP | DQ448218 |
| PN 54 | 32 | 10.29 | EP-AG-XI-KO-N-M-S-B-K | EP536827 |
| PN 55 | 1 | 0.32 | EP | EP536828 |
| PN 56 | 1 | 0.32 | EP | EP536829 |
| PN 57 | 1 | 0.32 | AG | EP536834 |
| PN 58 | 1 | 0.32 | XI | EP536846 |
| PN 59 | 1 | 0.32 | N | HM998866 |
| PN 60 | 2 | 0.64 | M | HM998862 |
| PN 61 | 1 | 0.32 | M | HM998865 |
| PN 62 | 1 | 0.32 | S | HM998863 |
