# Supplementary material for: Mitochondrial DNA Reveals Genetic Structuring of Pinna nobilis across the Mediterranean Sea
Source: PLoS One. 2013 Jun 28;8(6):e67372. doi: 10.1371/journal.pone.0067372 (PMC3696058; doi:10.1371/journal.pone.0067372)
Supplement: Table S2 — COI, COI-16S, 16S datasets: genetic divergence estimates. Sample sizes and genetic diversity estimates obtained for the mitochondrial regions analysed in Pinna nobilis. N: sample sizes; S: number of polymorphic sites; H: number of haplotypes; h: haplotype diversity; π: nucleotide diversity; d: mean of pairwise nucleotide differences. Populations are labelled as in Table 1. Sites with gaps were not considered. (DOC) [file pone.0067372.s004.doc]

| **Sample** | **N** | **S** | **H** | ***h*** | ***π*** | **d** |
| --- | --- | --- | --- | --- | --- | --- |
| **COI** | | | | | | |
| BPC | 18 | 10 | 7 | 0.725 | 0.005 | 1.667 |
| POR | 3 | 3 | 2 | 0.667 | 0.006 | 2.000 |
| LAZ | 2 | 4 | 2 | 1.000 | 0.012 | 4.000 |
| OSM | 21 | 9 | 8 | 0.829 | 0.006 | 1.886 |
| MOL | 11 | 6 | 6 | 0.873 | 0.006 | 1.964 |
| CCE | 13 | 5 | 5 | 0.705 | 0.005 | 1.615 |
| SAL | 5 | 3 | 3 | 0.700 | 0.003 | 1.200 |
| MPE | 4 | 5 | 3 | 0.833 | 0.007 | 2.500 |
| OTT | 5 | 6 | 4 | 0.900 | 0.008 | 2.800 |
| ORI | 10 | 8 | 7 | 0.911 | 0.007 | 2.356 |
| MAR | 5 | 7 | 4 | 0.900 | 0.009 | 3.000 |
| IMV | 4 | 0 | 1 | 0.000 | 0.000 | 0.000 |
| VMS | 4 | 5 | 4 | 1.000 | 0.008 | 2.833 |
| CPA | 5 | 4 | 3 | 0.700 | 0.005 | 1.800 |
| MAD | 18 | 11 | 10 | 0.895 | 0.007 | 2.242 |
| IPI | 13 | 12 | 9 | 0.949 | 0.009 | 3.205 |
| CPC | 12 | 7 | 6 | 0.803 | 0.005 | 1.803 |
| ELB | 10 | 7 | 6 | 0.889 | 0.008 | 2.822 |
| SVC | 7 | 5 | 4 | 0.714 | 0.005 | 1.714 |
| MON | 11 | 6 | 6 | 0.836 | 0.007 | 2.309 |
| MLZ | 10 | 6 | 5 | 0.867 | 0.007 | 2.533 |
| PAC | 8 | 8 | 7 | 0.964 | 0.007 | 2.536 |
| OGN | 15 | 7 | 9 | 0.886 | 0.007 | 2.362 |
| VEN | 20 | 10 | 10 | 0.895 | 0.006 | 2.184 |
| CYP | 2 | 1 | 2 | 1.000 | 0.003 | 1.000 |
| EP*^1^ | 9 | 9 | 6 | 0.833 | 0.007 | 2.500 |
| AG*^1^ | 9 | 2 | 3 | 0.667 | 0.002 | 0.778 |
| XI*^1^ | 5 | 2 | 3 | 0.700 | 0.002 | 0.800 |
| KO*^1^ | 3 | 0 | 1 | 0.000 | 0.000 | 0.000 |
| N*^2^ | 7 | 2 | 3 | 0.667 | 0.002 | 0.857 |
| M*^2^ | 9 | 4 | 4 | 0.694 | 0.004 | 1.389 |
| S*^2^ | 7 | 2 | 3 | 0.667 | 0.002 | 0.857 |
| B*^2^ | 9 | 1 | 2 | 0.556 | 0.002 | 0.556 |
| K*^2^ | 17 | 1 | 2 | 0.382 | 0.001 | 0.382 |
| **TOT** | 311 | 45 | 62 | 0.910 | 0.007 | 2.546 |
| **COI-16S** | | | | | | |
| BPC | 8 | 6 | 6 | 0.893 | 0.00231 | 1.821 |
| POR | 2 | 1 | 2 | 1.000 | 0.00127 | 1.000 |
| LAZ | 2 | 7 | 2 | 1.000 | 0.00888 | 7.000 |
| OSM | 20 | 12 | 10 | 0.874 | 0.00297 | 2.342 |
| MOL | 10 | 9 | 8 | 0.956 | 0.00403 | 3.178 |
| CCE | 12 | 7 | 7 | 0.833 | 0.00275 | 2.167 |
| SAL | 5 | 4 | 4 | 0.900 | 0.00203 | 1.600 |
| MPE | 4 | 7 | 3 | 0.833 | 0.00444 | 3.500 |
| OTT | 5 | 8 | 5 | 1.000 | 0.00482 | 3.800 |
| ORI | 9 | 10 | 6 | 0.889 | 0.00367 | 2.889 |
| MAR | 5 | 8 | 5 | 1.000 | 0.00431 | 3.400 |
| IMV | 4 | 3 | 3 | 0.833 | 0.00212 | 1.667 |
| VMS | 4 | 7 | 4 | 1.000 | 0.00508 | 4.000 |
| CPA | 5 | 8 | 5 | 1.000 | 0.00431 | 3.400 |
| MAD | 18 | 17 | 14 | 0.961 | 0.00392 | 3.092 |
| IPI | 13 | 16 | 10 | 0.962 | 0.00514 | 4.051 |
| CPC | 12 | 11 | 7 | 0.864 | 0.00313 | 2.470 |
| ELB | 10 | 12 | 8 | 0.956 | 0.00575 | 4.533 |
| SVC | 6 | 4 | 3 | 0.600 | 0.00195 | 1.533 |
| MON | 11 | 14 | 9 | 0.945 | 0.00519 | 4.091 |
| MLZ | 9 | 8 | 8 | 0.972 | 0.00458 | 3.611 |
| PAC | 8 | 11 | 8 | 1.000 | 0.00440 | 3.464 |
| OGN | 15 | 14 | 9 | 0.886 | 0.00463 | 3.648 |
| VEN | 20 | 16 | 12 | 0.921 | 0.00375 | 2.953 |
| CYP | 2 | 1 | 2 | 1.000 | 0.00127 | 1.000 |
| EP*^1^ | 8 | 8 | 5 | 0.786 | 0.00300 | 2.357 |
| AG*^1^ | 9 | 2 | 3 | 0.667 | 0.00099 | 0.778 |
| XI*^1^ | 5 | 2 | 3 | 0.700 | 0.00102 | 0.800 |
| KO*^1^ | 3 | 0 | 1 | 0.000 | 0.00000 | 0.000 |
| **TOT** | 244 | 233 | 172 | 0.949 | 0.00492 | 3.866 |
| **16S** | | | | | | |
| BPC | 9 | 4 | 4 | 0.583 | 0.00198 | 0.889 |
| POR | 2 | 1 | 2 | 1.000 | 0.00222 | 1.000 |
| LAZ | 2 | 3 | 2 | 1.000 | 0.00667 | 3.000 |
| OSM | 21 | 3 | 4 | 0.271 | 0.00083 | 0.371 |
| MOL | 10 | 3 | 4 | 0.778 | 0.00247 | 1.111 |
| CCE | 12 | 2 | 3 | 0.439 | 0.00104 | 0.470 |
| SAL | 5 | 1 | 1 | 0.400 | 0.00089 | 0.400 |
| MPE | 4 | 2 | 2 | 0.500 | 0.00222 | 1.000 |
| OTT | 5 | 1 | 2 | 0.400 | 0.00089 | 0.400 |
| ORI | 10 | 3 | 3 | 0.378 | 0.00133 | 0.600 |
| MAR | 5 | 1 | 2 | 0.400 | 0.00089 | 0.400 |
| IMV | 4 | 3 | 3 | 0.833 | 0.00370 | 1.667 |
| VMS | 4 | 2 | 3 | 0.833 | 0.00260 | 1.167 |
| CPA | 5 | 4 | 4 | 0.900 | 0.00356 | 1.600 |
| MAD | 18 | 6 | 5 | 0.549 | 0.00189 | 0.850 |
| IPI | 15 | 4 | 3 | 0.362 | 0.00165 | 0.743 |
| CPC | 13 | 4 | 3 | 0.295 | 0.00137 | 0.615 |
| ELB | 10 | 5 | 6 | 0.788 | 0.00380 | 1.711 |
| SVC | 7 | 0 | 1 | 0.000 | 0.00000 | 0.000 |
| MON | 11 | 8 | 8 | 0.891 | 0.00396 | 1.782 |
| MLZ | 9 | 2 | 3 | 0.667 | 0.00210 | 0.944 |
| PAC | 8 | 3 | 3 | 0.607 | 0.00206 | 0.929 |
| OGN | 15 | 7 | 4 | 0.371 | 0.00286 | 1.286 |
| VEN | 20 | 6 | 6 | 0.477 | 0.00171 | 0.768 |
| CYP | 2 | 0 | 1 | 0.000 | 0.00000 | 0.000 |
| EP*^1^ | 8 | 0 | 1 | 0.000 | 0.00000 | 0.000 |
| AG*^1^ | 9 | 0 | 1 | 0.000 | 0.00000 | 0.000 |
| XI*^1^ | 5 | 0 | 1 | 0.000 | 0.00000 | 0.000 |
| KO*^1^ | 3 | 0 | 1 | 0.000 | 0.00000 | 0.000 |
| **TOT** | 251 | 78 | 86 | 0.674 | 0.00279 | 1.245 |

Asterisks (*) and superscript numbers identify samples whose sequences were taken from the GenBank database: (1) Katsares et al. [35]; (2) Rabaoui et al. [36].
