# Supplementary material for: Mitochondrial DNA Reveals Genetic Structuring of Pinna nobilis across the Mediterranean Sea
Source: PLoS One. 2013 Jun 28;8(6):e67372. doi: 10.1371/journal.pone.0067372 (PMC3696058; doi:10.1371/journal.pone.0067372)
Supplement: Table S3 — COI dataset: Bayesian COI haplogroup frequencies. N: absolute frequency; %: relative frequency within Mediterranean populations of Pinna nobilis. Populations are labelled as in Table 1. (DOC) [file pone.0067372.s005.doc]

|  | **P1** | | **P2** | | **P3** | | **P4** | |
| --- | --- | --- | --- | --- | --- | --- | --- | --- |
| **Sample** | N | % | N | % | N | % | N | % |
| BPC | 15 | 4.82 | 0 | 0.00 | 2 | 0.64 | 1 | 0.32 |
| POR | 2 | 0.64 | 0 | 0.00 | 0 | 0.00 | 1 | 0.32 |
| LAZ | 1 | 0.32 | 0 | 0.00 | 0 | 0.00 | 1 | 0.32 |
| OSM | 13 | 4.18 | 0 | 0.00 | 7 | 2.25 | 1 | 0.32 |
| MOL | 6 | 1.93 | 0 | 0.00 | 0 | 0.00 | 5 | 1.61 |
| CCE | 10 | 3.22 | 0 | 0.00 | 3 | 0.96 | 0 | 0.00 |
| SAL | 1 | 0.32 | 0 | 0.00 | 4 | 1.29 | 0 | 0.00 |
| MPE | 3 | 0.96 | 0 | 0.00 | 1 | 0.32 | 0 | 0.00 |
| OTT | 2 | 0.64 | 0 | 0.00 | 2 | 0.64 | 1 | 0.32 |
| ORI | 5 | 1.61 | 0 | 0.00 | 5 | 1.61 | 0 | 0.00 |
| MAR | 1 | 0.32 | 0 | 0.00 | 4 | 1.29 | 0 | 0.00 |
| IMV | 0 | 0.00 | 0 | 0.00 | 4 | 1.29 | 0 | 0.00 |
| VMS | 2 | 0.64 | 0 | 0.00 | 2 | 0.64 | 0 | 0.00 |
| CPA | 4 | 1.29 | 0 | 0.00 | 0 | 0.00 | 1 | 0.32 |
| MAD | 13 | 4.18 | 0 | 0.00 | 4 | 1.29 | 1 | 0.32 |
| IPI | 8 | 2.57 | 0 | 0.00 | 2 | 0.64 | 3 | 0.96 |
| CPC | 9 | 2.89 | 0 | 0.00 | 2 | 0.64 | 1 | 0.32 |
| ELB | 3 | 0.96 | 0 | 0.00 | 4 | 1.29 | 3 | 0.96 |
| SVC | 6 | 1.93 | 0 | 0.00 | 1 | 0.32 | 0 | 0.00 |
| MON | 5 | 1.61 | 0 | 0.00 | 5 | 1.61 | 1 | 0.32 |
| MLZ | 3 | 0.96 | 0 | 0.00 | 5 | 1.61 | 2 | 0.64 |
| PAC | 5 | 1.61 | 0 | 0.00 | 2 | 0.64 | 1 | 0.32 |
| OGN | 11 | 3.54 | 0 | 0.00 | 4 | 1.29 | 0 | 0.00 |
| VEN | 2 | 0.64 | 0 | 0.00 | 16 | 5.14 | 2 | 0.64 |
| CYP | 0 | 0.00 | 0 | 0.00 | 2 | 0.64 | 0 | 0.00 |
| EP*^1^ | 0 | 0.00 | 8 | 2.57 | 1 | 0.32 | 0 | 0.00 |
| AG*^1^ | 0 | 0.00 | 9 | 2.89 | 0 | 0.00 | 0 | 0.00 |
| XI*^1^ | 0 | 0.00 | 5 | 1.61 | 0 | 0.00 | 0 | 0.00 |
| KO*^1^ | 0 | 0.00 | 3 | 0.96 | 0 | 0.00 | 0 | 0.00 |
| N*^2^ | 0 | 0.00 | 7 | 2.25 | 0 | 0.00 | 0 | 0.00 |
| M*^2^ | 0 | 0.00 | 8 | 2.57 | 1 | 0.32 | 0 | 0.00 |
| S*^2^ | 0 | 0.00 | 7 | 2.25 | 0 | 0.00 | 0 | 0.00 |
| B*^2^ | 0 | 0.00 | 9 | 2.89 | 0 | 0.00 | 0 | 0.00 |
| K*^2^ | 0 | 0.00 | 17 | 5.47 | 0 | 0.00 | 0 | 0.00 |
| **TOT** | 130 | 41.80 | 73 | 23.47 | 83 | 26.69 | 25 | 8.04 |

Asterisks (*) and superscript numbers identify samples whose sequences were taken from the GenBank database: (1) Katsares et al. [35]; (2) Rabaoui et al. [36].
