# Supplementary material for: Mitochondrial DNA Reveals Genetic Structuring of Pinna nobilis across the Mediterranean Sea
Source: PLoS One. 2013 Jun 28;8(6):e67372. doi: 10.1371/journal.pone.0067372 (PMC3696058; doi:10.1371/journal.pone.0067372)
Supplement: Table S4 — COI dataset: pairwise ΦST values among sampling localities. Pairwise ΦST values between Pinna nobilis populations with at least five individuals. Significance was assessed by permutation test. Significant values after correction for multiple testing are reported in bold. Populations are labelled as in Table 1. (DOC) [file pone.0067372.s006.doc]

|  | **BMC** | **OSM** | **MOL** | **CCE** | **SAL** | **OTT** | **ORI** | **MAR** | **CPA** | **MAD** | **IPI** | **CPC** | **ELB** | **SVC** | **MON** | **MLZ** | **PAC** | **OGN** | **VEN** | **EP*^1^** | **AG*^1^** | **XI*^1^** | **M*^2^** | **N*^2^** | **S*^2^** | **B*^2^** | **K*^2^** |
| --- | --- | --- | --- | --- | --- | --- | --- | --- | --- | --- | --- | --- | --- | --- | --- | --- | --- | --- | --- | --- | --- | --- | --- | --- | --- | --- | --- |
| **BMC** | 0 |  |  |  |  |  |  |  |  |  |  |  |  |  |  |  |  |  |  |  |  |  |  |  |  |  |  |
| **OSM** | 0.021 | 0 |  |  |  |  |  |  |  |  |  |  |  |  |  |  |  |  |  |  |  |  |  |  |  |  |  |
| **MOL** | **0.160** | **0.237** | 0 |  |  |  |  |  |  |  |  |  |  |  |  |  |  |  |  |  |  |  |  |  |  |  |  |
| **CCE** | -0.008 | -0.003 | **0.265** | 0 |  |  |  |  |  |  |  |  |  |  |  |  |  |  |  |  |  |  |  |  |  |  |  |
| **SAL** | **0.374** | 0.161 | **0.514** | **0.336** | 0 |  |  |  |  |  |  |  |  |  |  |  |  |  |  |  |  |  |  |  |  |  |  |
| **OTT** | -0.011 | -0.076 | 0.095 | -0.002 | 0.082 | 0 |  |  |  |  |  |  |  |  |  |  |  |  |  |  |  |  |  |  |  |  |  |
| **ORI** | 0.064 | -0.022 | **0.251** | 0.063 | 0.073 | -0.071 | 0 |  |  |  |  |  |  |  |  |  |  |  |  |  |  |  |  |  |  |  |  |
| **MAR** | **0.344** | 0.180 | **0.445** | 0.280 | -0.135 | 0.040 | 0.075 | 0 |  |  |  |  |  |  |  |  |  |  |  |  |  |  |  |  |  |  |  |
| **CPA** | -0.021 | 0.100 | 0.023 | 0.012 | **0.516** | 0.004 | 0.156 | 0.366 | 0 |  |  |  |  |  |  |  |  |  |  |  |  |  |  |  |  |  |  |
| **MAD** | -0.018 | -0.002 | **0.182** | -0.045 | **0.250** | -0.037 | 0.021 | **0.219** | -0.026 | 0 |  |  |  |  |  |  |  |  |  |  |  |  |  |  |  |  |  |
| **IPI** | -0.014 | 0.045 | 0.042 | -0.001 | **0.266** | -0.050 | 0.080 | **0.240** | -0.120 | -0.006 | 0 |  |  |  |  |  |  |  |  |  |  |  |  |  |  |  |  |
| **CPC** | -0.049 | -0.011 | 0.144 | -0.050 | **0.340** | -0.052 | 0.042 | **0.287** | -0.065 | -0.053 | -0.047 | 0 |  |  |  |  |  |  |  |  |  |  |  |  |  |  |  |
| **ELB** | 0.065 | 0.023 | 0.065 | 0.085 | 0.150 | -0.132 | 0.021 | 0.110 | 0.037 | 0.036 | 0.004 | 0.015 | 0 |  |  |  |  |  |  |  |  |  |  |  |  |  |  |
| **SVC** | -0.033 | 0.020 | **0.226** | -0.071 | **0.412** | 0.006 | 0.076 | 0.301 | -0.045 | -0.056 | -0.034 | -0.078 | 0.069 | 0 |  |  |  |  |  |  |  |  |  |  |  |  |  |
| **MON** | 0.074 | -0.033 | **0.261** | 0.000 | 0.080 | -0.098 | -0.004 | 0.068 | 0.111 | 0.006 | 0.041 | 0.008 | -0.008 | 0.029 | 0 |  |  |  |  |  |  |  |  |  |  |  |  |
| **MLZ** | **0.159** | 0.039 | **0.216** | 0.163 | 0.006 | -0.099 | -0.018 | 0.003 | 0.201 | 0.102 | 0.107 | 0.112 | -0.058 | 0.172 | 0.000 | 0 |  |  |  |  |  |  |  |  |  |  |  |
| **PAC** | -0.042 | -0.057 | 0.109 | -0.031 | 0.183 | -0.128 | -0.035 | 0.153 | -0.036 | -0.046 | -0.043 | -0.075 | -0.046 | -0.029 | -0.046 | 0.006 | 0 |  |  |  |  |  |  |  |  |  |  |
| **OGN** | 0.037 | 0.013 | **0.237** | -0.012 | 0.212 | -0.033 | 0.041 | 0.175 | 0.052 | -0.008 | 0.034 | -0.024 | 0.022 | -0.048 | -0.031 | 0.075 | -0.015 | 0 |  |  |  |  |  |  |  |  |  |
| **VEN** | **0.391** | **0.235** | **0.481** | **0.349** | -0.100 | 0.143 | **0.161** | -0.048 | **0.455** | **0.308** | **0.341** | **0.347** | **0.188** | **0.382** | **0.154** | 0.052 | **0.247** | **0.260** | 0 |  |  |  |  |  |  |  |  |
| **EP*^1^** | **0.379** | **0.393** | **0.426** | **0.351** | **0.519** | **0.296** | **0.401** | **0.461** | **0.296** | **0.331** | **0.276** | **0.343** | **0.339** | **0.306** | **0.359** | **0.414** | **0.315** | **0.340** | **0.544** | 0 |  |  |  |  |  |  |  |
| **AG*^1^** | **0.415** | **0.432** | **0.506** | **0.461** | **0.738** | **0.452** | **0.469** | **0.628** | **0.494** | **0.374** | **0.319** | **0.430** | **0.424** | **0.470** | **0.465** | **0.500** | **0.403** | **0.409** | **0.611** | 0.037 | 0 |  |  |  |  |  |  |
| **XI*^1^** | **0.431** | **0.457** | **0.518** | **0.439** | **0.755** | **0.434** | **0.485** | **0.596** | **0.445** | **0.362** | **0.286** | **0.426** | **0.420** | **0.430** | **0.451** | **0.513** | **0.400** | **0.389** | **0.625** | -0.054 | 0.071 | 0 |  |  |  |  |  |
| **M*^2^** | **0.400** | **0.409** | **0.499** | **0.420** | **0.712** | **0.404** | **0.442** | **0.579** | **0.457** | **0.342** | **0.285** | **0.401** | **0.393** | **0.425** | **0.419** | **0.470** | **0.369** | **0.372** | **0.584** | -0.037 | -0.069 | -0.033 | 0 |  |  |  |  |
| **N*^2^** | **0.386** | **0.397** | **0.448** | **0.400** | **0.638** | **0.356** | **0.425** | **0.541** | **0.387** | **0.337** | **0.277** | **0.375** | **0.355** | **0.388** | **0.390** | **0.437** | **0.345** | **0.357** | **0.565** | -0.009 | -0.022 | -0.008 | -0.084 | 0 |  |  |  |
| **S*^2^** | **0.410** | **0.427** | **0.490** | **0.435** | **0.727** | **0.423** | **0.462** | **0.601** | **0.440** | **0.356** | **0.286** | **0.409** | **0.408** | **0.438** | **0.440** | **0.490** | **0.376** | **0.391** | **0.607** | -0.016 | -0.078 | -0.034 | -0.107 | -0.057 | 0 |  |  |
| **B*^2^** | **0.423** | **0.437** | **0.519** | **0.475** | **0.769** | **0.473** | **0.482** | **0.648** | **0.528** | **0.379** | **0.323** | **0.444** | **0.433** | **0.496** | **0.476** | **0.511** | **0.417** | **0.417** | **0.616** | 0.032 | -0.102 | 0.095 | -0.080 | -0.032 | -0.087 | 0 |  |
| **K*^2^** | **0.490** | **0.489** | **0.589** | **0.571** | **0.817** | **0.581** | **0.551** | **0.736** | **0.654** | **0.460** | **0.422** | **0.533** | **0.616** | **0.564** | **0.577** | **0.511** | **0.505** | **0.517** | **0.657** | 0.203 | 0.023 | 0.391 | 0.138 | 0.128 | 0.121 | 0.014 | 0 |

Asterisks (*) and superscript numbers identify samples whose sequences were taken from the GenBank database: (1) Katsares et al. [35]; (2) Rabaoui et al. [36]
