# Supplementary material for: Mitochondrial DNA Reveals Genetic Structuring of Pinna nobilis across the Mediterranean Sea
Source: PLoS One. 2013 Jun 28;8(6):e67372. doi: 10.1371/journal.pone.0067372 (PMC3696058; doi:10.1371/journal.pone.0067372)
Supplement: Table S5 — COI-16S dataset: haplotype frequencies. Frequency distribution of mitochondrial haplotypes in 244 individuals from 29 populations of Pinna nobilis. N: absolute frequency; %: relative frequency within Mediterranean populations. Populations are labelled as in Table 1. (DOC) [file pone.0067372.s007.doc]

| **Clade** | **N** | **%** | **Populations** |
| --- | --- | --- | --- |
| PNCS 1 | 23 | 9.43 | BMC-OSM-MOL-CCE-OTT-ORI-MAD- ELB- SVC-MLZ-PAC-OGN |
| PNCS 2 | 1 | 0.41 | BMC |
| PNCS 3 | 1 | 0.41 | BMC |
| PNCS 4 | 1 | 0.41 | BMC |
| PNCS 5 | 1 | 0.41 | BMC |
| PNCS 6 | 35 | 14.34 | BMC-POR-OSM-MOL-CCE-MPE-VSM-CPA-MAD-ELB- SVC-MON-PAC-OGN |
| PNCS 7 | 1 | 0.41 | POR |
| PNCS 8 | 2 | 0.82 | LAZ-OTT |
| PNCS 9 | 1 | 0.41 | LAZ |
| PNCS 10 | 13 | 5.33 | OSM-SAL-MAR-MAD-ELB-MLZ-OGN |
| PNCS 11 | 1 | 0.41 | OSM |
| PNCS 12 | 1 | 0.41 | OSM |
| PNCS 13 | 1 | 0.41 | OSM |
| PNCS 14 | 7 | 2.87 | OSM-MOL-CPA-MAD-ELB-MLZ-PAC |
| PNCS 15 | 3 | 1.23 | OSM-CCE-MPE |
| PNCS 16 | 7 | 2.87 | OSM-MOL-CCE-CPA-MAD-MON-PAC |
| PNCS 17 | 1 | 0.41 | OSM |
| PNCS 18 | 6 | 2.46 | MOL-SAL-OTT-VSM-VEN |
| PNCS 19 | 3 | 1.23 | MOL-MLZ |
| PNCS 20 | 1 | 0.41 | MOL |
| PNCS 21 | 1 | 0.41 | MOL |
| PNCS 22 | 1 | 0.41 | CCE |
| PNCS 23 | 9 | 3.69 | CCE-SAL-OTT-VEN |
| PNCS 24 | 5 | 2.05 | CCE-MAR-MAD-MON-PAC |
| PNCS 25 | 1 | 0.41 | MPE |
| PNCS 26 | 5 | 2.05 | SAL-IMV-VEN |
| PNCS 27 | 1 | 0.41 | OTT |
| PNCS 28 | 1 | 0.41 | ORI |
| PNCS 29 | 2 | 0.82 | ORI |
| PNCS 30 | 1 | 0.41 | ORI |
| PNCS 31 | 1 | 0.41 | ORI |
| PNCS 32 | 1 | 0.41 | ORI |
| PNCS 33 | 1 | 0.41 | MAR |
| PNCS 34 | 1 | 0.41 | MAR |
| PNCS 35 | 1 | 0.41 | MAR |
| PNCS 36 | 3 | 1.23 | IMV-MLZ |
| PNCS 37 | 1 | 0.41 | IMV |
| PNCS 38 | 1 | 0.41 | VSM |
| PNCS 39 | 1 | 0.41 | VSM |
| PNCS 40 | 1 | 0.41 | CPA |
| PNCS 41 | 1 | 0.41 | CPA |
| PNCS 42 | 1 | 0.41 | MAD |
| PNCS 43 | 1 | 0.41 | MAD |
| PNCS 44 | 1 | 0.41 | MAD |
| PNCS 45 | 1 | 0.41 | MAD |
| PNCS 46 | 1 | 0.41 | MAD |
| PNCS 47 | 1 | 0.41 | MAD |
| PNCS 48 | 3 | 1.23 | MAD-MLZ-OGN |
| PNCS 49 | 1 | 0.41 | MAD |
| PNCS 50 | 1 | 0.41 | IPI |
| PNCS 51 | 4 | 1.64 | IPI-CPC-CYP |
| PNCS 52 | 6 | 2.46 | IPI-CPC |
| PNCS 53 | 1 | 0.41 | IPI |
| PNCS 54 | 3 | 1.23 | IPI-CPC |
| PNCS 55 | 1 | 0.41 | IPI |
| PNCS 56 | 1 | 0.41 | IPI |
| PNCS 57 | 1 | 0.41 | IPI |
| PNCS 58 | 1 | 0.41 | IPI |
| PNCS 59 | 1 | 0.41 | IPI |
| PNCS 60 | 1 | 0.41 | CPC |
| PNCS 61 | 1 | 0.41 | CPC |
| PNCS 62 | 3 | 1.23 | CPC |
| PNCS 63 | 1 | 0.41 | CPC |
| PNCS 64 | 1 | 0.41 | SVC |
| PNCS 65 | 1 | 0.41 | MON |
| PNCS 66 | 1 | 0.41 | MON |
| PNCS 67 | 1 | 0.41 | MON |
| PNCS 68 | 1 | 0.41 | MON |
| PNCS 69 | 1 | 0.41 | MON |
| PNCS 70 | 1 | 0.41 | MON |
| PNCS 71 | 1 | 0.41 | MLZ |
| PNCS 72 | 1 | 0.41 | MLZ |
| PNCS 73 | 1 | 0.41 | PAC |
| PNCS 74 | 1 | 0.41 | PAC |
| PNCS 75 | 1 | 0.41 | PAC |
| PNCS 76 | 1 | 0.41 | OGN |
| PNCS 77 | 1 | 0.41 | OGN |
| PNCS 78 | 1 | 0.41 | OGN |
| PNCS 79 | 1 | 0.41 | OGN |
| PNCS 80 | 2 | 0.82 | OGN |
| PNCS 81 | 1 | 0.41 | ELB |
| PNCS 82 | 1 | 0.41 | ELB |
| PNCS 83 | 1 | 0.41 | ELB |
| PNCS 84 | 1 | 0.41 | ELB |
| PNCS 85 | 1 | 0.41 | VEN |
| PNCS 86 | 1 | 0.41 | VEN |
| PNCS 87 | 1 | 0.41 | VEN |
| PNCS 88 | 1 | 0.41 | VEN |
| PNCS 89 | 2 | 0.82 | VEN |
| PNCS 90 | 1 | 0.41 | VEN |
| PNCS 91 | 1 | 0.41 | VEN |
| PNCS 92 | 1 | 0.41 | VEN |
| PNCS 93 | 1 | 0.41 | VEN |
| PNCS 94 | 1 | 0.41 | CYP |
| PNCS 95 | 1 | 0.41 | EP |
| PNCS 96 | 8 | 3.28 | EP-AG |
| PNCS 97 | 8 | 3.28 | EP-AG-KO |
| PNCS 98 | 1 | 0.41 | EP |
| PNCS 99 | 1 | 0.41 | EP |
| PNCS 100 | 1 | 0.41 | AG |
| PNCS 101 | 3 | 1.23 | XI |
| PNCS 102 | 1 | 0.41 | XI |
| PNCS 103 | 1 | 0.41 | XI |
