# Supplementary material for: Mitochondrial DNA Reveals Genetic Structuring of Pinna nobilis across the Mediterranean Sea
Source: PLoS One. 2013 Jun 28;8(6):e67372. doi: 10.1371/journal.pone.0067372 (PMC3696058; doi:10.1371/journal.pone.0067372)
Supplement: Table S6 — 16S dataset: haplotype frequencies. Frequency distribution of haplotypes in 251 individuals from 29 populations of Pinna nobilis. N: absolute frequency; %: relative frequency within Mediterranean populations. Populations are labelled as in Table 1. (DOC) [file pone.0067372.s008.doc]

| **Clade** | **N** | **%** | **Populations** | **# GenBank** |
| --- | --- | --- | --- | --- |
| PNS 1 | 108 | 43.03 | BMC-POR-OSM-MOL-CCE-MPE-SAL-OTT-CPA-ORI-MAR-VSM-MAD-ELB-SVC-MON-MLZ-PAC-OGN | JX854562 |
| PNS 2 | 1 | 0.40 | BMC | JX854566 |
| PNS 3 | 10 | 3.98 | BMC-OSM-MOL-CPA-MAD-ELB-MLZ-PAC | JX854569 |
| PNS 4 | 1 | 0.40 | BMC | JX854570 |
| PNS 5 | 12 | 4.78 | POR-LAZ-OSM-MOL-OTT-IMV-MON-MLZ | JX854572 |
| PNS 6 | 1 | 0.40 | LAZ | JX854574 |
| PNS 7 | 1 | 0.40 | OSM | JX854580 |
| PNS 8 | 28 | 11.16 | MOL-CCE-SAL-OTT-IMV-VSM-VEN | JX854596 |
| PNS 9 | 10 | 3.98 | CCE-MAR-MAD-ELB-MON-PAC-VEN | JX854616 |
| PNS 10 | 1 | 0.40 | MPE | JX854618 |
| PNS 11 | 1 | 0.40 | CPA | JX854634 |
| PNS 12 | 1 | 0.40 | CPA | JX854636 |
| PNS 13 | 1 | 0.40 | ORI | JX854639 |
| PNS 14 | 2 | 0.80 | ORI-MON | JX854645 |
| PNS 15 | 1 | 0.40 | IMV | JX854655 |
| PNS 16 | 1 | 0.40 | VSM | JX854656 |
| PNS 17 | 1 | 0.40 | MAD | JX854660 |
| PNS 18 | 1 | 0.40 | MAD | JX854666 |
| PNS 19 | 24 | 9.56 | IPI-CPC-CYP | JX854678 |
| PNS 20 | 2 | 0.80 | IPI | JX854687 |
| PNS 21 | 1 | 0.40 | IPI | JX854692 |
| PNS 22 | 1 | 0.40 | CPC | JX854694 |
| PNS 23 | 1 | 0.40 | CPC | JX854702 |
| PNS 24 | 1 | 0.40 | MON | JX854713 |
| PNS 25 | 1 | 0.40 | MON | JX854715 |
| PNS 26 | 1 | 0.40 | MON | JX854718 |
| PNS 27 | 1 | 0.40 | MON | JX854719 |
| PNS 28 | 1 | 0.40 | OGN | JX854748 |
| PNS 29 | 1 | 0.40 | OGN | JX854749 |
| PNS 30 | 1 | 0.40 | OGN | JX854750 |
| PNS 31 | 1 | 0.40 | ELB | JX854757 |
| PNS 32 | 1 | 0.40 | ELB | JX854762 |
| PNS 33 | 1 | 0.40 | ELB | JX854765 |
| PNS 34 | 1 | 0.40 | VEN | JX854767 |
| PNS 35 | 1 | 0.40 | VEN | JX854768 |
| PNS 36 | 1 | 0.40 | VEN | JX854769 |
| PNS 37 | 1 | 0.40 | VEN | JX854771 |
| PNS 38 | 1 | 0.40 | CYP | JX854787 |
| PNS 39 | 5 | 1.99 | XI | EF536855 |
| PNS 40 | 20 | 7.97 | EP-AG-KO | DQ663473 |
