# Supplementary material for: Mitochondrial DNA Reveals Genetic Structuring of Pinna nobilis across the Mediterranean Sea
Source: PLoS One. 2013 Jun 28;8(6):e67372. doi: 10.1371/journal.pone.0067372 (PMC3696058; doi:10.1371/journal.pone.0067372)
Supplement: Table S7 — COI-16S dataset: Bayesian COI-16S mitochondrial region haplogroup frequencies. N: absolute frequency; %: relative frequency within Mediterranean populations of Pinna nobilis. Populations are labelled as in Table 1. (DOC) [file pone.0067372.s009.doc]

|  | **N1** | | **N2** | | **N3** | | **N4** | |
| --- | --- | --- | --- | --- | --- | --- | --- | --- |
| **Sample** | N | % | N | % | N | % | N | % |
| BPC | 0 | 0.00 | 8 | 3.28 | 0 | 0.00 | 0 | 0.00 |
| POR | 0 | 0.00 | 2 | 0.82 | 0 | 0.00 | 0 | 0.00 |
| LAZ | 1 | 0.41 | 1 | 0.41 | 0 | 0.00 | 0 | 0.00 |
| OSM | 1 | 0.41 | 12 | 4.92 | 7 | 2.87 | 0 | 0.00 |
| MOL | 5 | 2.05 | 5 | 2.05 | 0 | 0.00 | 0 | 0.00 |
| CCE | 0 | 0.00 | 9 | 3.69 | 3 | 1.23 | 0 | 0.00 |
| SAL | 0 | 0.00 | 3 | 1.23 | 1 | 0.41 | 0 | 0.00 |
| MPE | 0 | 0.00 | 1 | 0.41 | 4 | 1.64 | 0 | 0.00 |
| OTT | 1 | 0.41 | 2 | 0.82 | 2 | 0.82 | 0 | 0.00 |
| ORI | 0 | 0.00 | 5 | 2.05 | 4 | 1.64 | 0 | 0.00 |
| MAR | 0 | 0.00 | 1 | 0.41 | 4 | 1.64 | 0 | 0.00 |
| IMV | 0 | 0.00 | 0 | 0.00 | 4 | 1.64 | 0 | 0.00 |
| VMS | 0 | 0.00 | 2 | 0.82 | 2 | 0.82 | 0 | 0.00 |
| CPA | 1 | 0.41 | 4 | 1.64 | 0 | 0.00 | 0 | 0.00 |
| MAD | 1 | 0.41 | 13 | 5.33 | 4 | 1.64 | 0 | 0.00 |
| IPI | 3 | 1.23 | 8 | 3.28 | 2 | 0.82 | 0 | 0.00 |
| CPC | 1 | 0.41 | 9 | 3.69 | 2 | 0.82 | 0 | 0.00 |
| ELB | 3 | 1.23 | 3 | 1.23 | 4 | 1.64 | 0 | 0.00 |
| SVC | 0 | 0.00 | 5 | 2.05 | 1 | 0.41 | 0 | 0.00 |
| MON | 1 | 0.41 | 5 | 2.05 | 5 | 2.05 | 0 | 0.00 |
| MLZ | 2 | 0.82 | 2 | 0.82 | 5 | 2.05 | 0 | 0.00 |
| PAC | 1 | 0.41 | 5 | 2.05 | 2 | 0.82 | 0 | 0.00 |
| OGN | 0 | 0.00 | 10 | 4.10 | 5 | 2.05 | 0 | 0.00 |
| VEN | 1 | 0.41 | 2 | 0.82 | 17 | 6.97 | 0 | 0.00 |
| CYP | 0 | 0.00 | 0 | 0.00 | 2 | 0.82 | 0 | 0.00 |
| EP* | 0 | 0.00 | 0 | 0.00 | 0 | 0.00 | 8 | 3.28 |
| AG* | 0 | 0.00 | 0 | 0.00 | 0 | 0.00 | 9 | 3.69 |
| XI* | 0 | 0.00 | 5 | 2.05 | 0 | 0.00 | 0 | 0.00 |
| KO* | 0 | 0.00 | 0 | 0.00 | 0 | 0.00 | 3 | 1.23 |
| **TOT** | 22 | 9.02 | 122 | 50.00 | 80 | 32.79 | 20 | 8.20 |

Asterisks (*) identify samples whose sequences were taken from the GenBank database: Katsares et al. [35].
