# Supplementary material for: Mitochondrial DNA Reveals Genetic Structuring of Pinna nobilis across the Mediterranean Sea
Source: PLoS One. 2013 Jun 28;8(6):e67372. doi: 10.1371/journal.pone.0067372 (PMC3696058; doi:10.1371/journal.pone.0067372)
Supplement: Table S8 — COI-16S dataset: pairwise ΦST values among samples localities. Pairwise ΦST values between Pinna nobilis populations with at least five individuals. Significance was assessed by permutation test. Significant values after correction for multiple testing are reported in bold. Populations are labelled as in Table 1. (DOC) [file pone.0067372.s010.doc]

|  | **BMC** | **OSM** | **MOL** | **CCE** | **SAL** | **OTT** | **ORI** | **MAR** | **CPA** | **MAD** | **IPI** | **CPC** | **ELB** | **SVC** | **MON** | **MLZ** | **PAC** | **OGN** | **VEN** | **EP*** | **AG*** | **XI*** |
| --- | --- | --- | --- | --- | --- | --- | --- | --- | --- | --- | --- | --- | --- | --- | --- | --- | --- | --- | --- | --- | --- | --- |
| **BMC** | 0 |  |  |  |  |  |  |  |  |  |  |  |  |  |  |  |  |  |  |  |  |  |
| **OSM** | 0.092 | 0 |  |  |  |  |  |  |  |  |  |  |  |  |  |  |  |  |  |  |  |  |
| **MOL** | 0.219 | **0.212** | 0 |  |  |  |  |  |  |  |  |  |  |  |  |  |  |  |  |  |  |  |
| **CCE** | 0.021 | -0.004 | **0.240** | 0 |  |  |  |  |  |  |  |  |  |  |  |  |  |  |  |  |  |  |
| **SAL** | 0.539 | **0.290** | **0.463** | **0.339** | 0 |  |  |  |  |  |  |  |  |  |  |  |  |  |  |  |  |  |
| **OTT** | 0.190 | 0.055 | 0.119 | 0.048 | 0.024 | 0 |  |  |  |  |  |  |  |  |  |  |  |  |  |  |  |  |
| **ORI** | 0.119 | -0.011 | **0.209** | 0.047 | **0.247** | 0.044 | 0 |  |  |  |  |  |  |  |  |  |  |  |  |  |  |  |
| **MAR** | 0.368 | 0.146 | **0.368** | 0.213 | 0.133 | 0.102 | 0.076 | 0 |  |  |  |  |  |  |  |  |  |  |  |  |  |  |
| **CPA** | -0.010 | 0.088 | 0.020 | 0.032 | **0.460** | 0.081 | 0.110 | 0.283 | 0 |  |  |  |  |  |  |  |  |  |  |  |  |  |
| **MAD** | -0.002 | 0.001 | **0.171** | -0.037 | **0.304** | 0.070 | 0.015 | 0.151 | -0.017 | 0 |  |  |  |  |  |  |  |  |  |  |  |  |
| **IPI** | 0.237 | **0.267** | **0.234** | **0.248** | **0.423** | **0.221** | **0.251** | **0.329** | 0.150 | **0.224** | 0 |  |  |  |  |  |  |  |  |  |  |  |
| **CPC** | 0.298 | **0.288** | **0.341** | **0.283** | **0.518** | **0.302** | **0.289** | **0.402** | **0.242** | **0.236** | -0.034 | 0 |  |  |  |  |  |  |  |  |  |  |
| **ELB** | 0.133 | 0.044 | 0.040 | 0.082 | **0.221** | -0.006 | 0.038 | 0.064 | 0.022 | 0.033 | **0.196** | **0.250** | 0 |  |  |  |  |  |  |  |  |  |
| **SVC** | -0.035 | 0.026 | 0.236 | -0.070 | **0.518** | 0.136 | 0.081 | 0.283 | -0.028 | -0.057 | **0.215** | **0.278** | -0.035 | 0 |  |  |  |  |  |  |  |  |
| **MON** | 0.115 | -0.006 | **0.148** | 0.026 | 0.189 | 0.001 | 0.003 | 0.038 | 0.039 | 0.019 | **0.208** | **0.244** | 0.020 | 0.115 | 0 |  |  |  |  |  |  |  |
| **MLZ** | **0.278** | 0.082 | 0.142 | **0.192** | 0.193 | 0.028 | 0.043 | 0.030 | 0.163 | **0.132** | **0.265** | **0.333** | 0.218 | -0.011 | **0.278** | 0 |  |  |  |  |  |  |
| **PAC** | 0.028 | -0.034 | 0.089 | -0.023 | **0.277** | 0.001 | -0.015 | 0.108 | -0.033 | -0.050 | **0.184** | **0.231** | -0.003 | -0.035 | 0.042 | 0.028 | 0 |  |  |  |  |  |
| **OGN** | 0.039 | 0.011 | **0.177** | -0.005 | **0.257** | 0.052 | 0.024 | 0.108 | 0.015 | -0.007 | **0.220** | **0.226** | -0.062 | -0.019 | 0.080 | -0.018 | 0.039 | 0 |  |  |  |  |
| **VEN** | **0.473** | **0.327** | **0.458** | **0.342** | -0.103 | 0.095 | **0.283** | **0.160** | **0.448** | **0.352** | **0.468** | **0.499** | **0.417** | **0.267** | **0.231** | **0.319** | **0.314** | **0.280** | 0 |  |  |  |
| **EP*** | **0.596** | **0.581** | **0.584** | **0.574** | **0.698** | **0.551** | **0.567** | **0.600** | **0.525** | **0.514** | **0.533** | **0.625** | **0.586** | **0.496** | **0.571** | **0.522** | **0.491** | **0.495** | **0.651** | 0 |  |  |
| **AG*** | **0.703** | **0.632** | **0.648** | **0.661** | **0.830** | **0.674** | **0.647** | **0.733** | **0.653** | **0.564** | **0.589** | **0.695** | **0.740** | **0.570** | **0.652** | **0.608** | **0.550** | **0.564** | **0.701** | 0.050 | 0 |  |
| **XI*** | **0.405** | **0.396** | **0.436** | **0.371** | **0.743** | **0.437** | **0.416** | **0.554** | **0.310** | **0.275** | **0.382** | **0.499** | **0.435** | **0.313** | **0.467** | **0.331** | **0.268** | **0.325** | **0.587** | **0.519** | **0.726** | 0 |

Asterisks (*) identify samples whose sequences were taken from the GenBank database: Katsares et al. [34].
